# Salinity-driven shifts in estuarine viral community composition and diversity near the Shenzhen coast

**DOI:** 10.1128/aem.00407-25

**Published:** 2025-07-02

**Authors:** Sarfraz Hussain, Xiaomeng Wang, Cong Pan, Songze Chen, Jiajia Xie, Nazia Mahtab, Shengwei Hou, Shuangfei Li

**Affiliations:** 1 Shenzhen Key Laboratory of Marine Bioresource & Eco-environmental Sciences, College of Life Sciences and Oceanography, Shenzhen University 47890, Shenzhen, China; 2 College of Physics and Optoelectronic Engineering, Shenzhen University 528555 , Shenzhen, China; 3 Department of Ocean Science and Engineering, Southern University of Science and Technology 547494 , Shenzhen, China; 4 Shenzhen Ecological Environment Monitoring Center Station, Shenzhen, China; Colorado School of Mines, Golden, Colorado, USA

**Keywords:** estuarine ecosystem, salinity, marine viruses, alginate lyase, auxiliary metabolic genes

## Abstract

Estuarine viral communities play a key role in microbial dynamics and ecosystem functioning. However, how viruses adapt to the highly dynamic estuarine environments remains largely underexplored. This study uses viromic sequencing to investigate the DNA viruses in estuarine water samples adjacent to the Shenzhen coast. Samples were divided into two major groups based on variations in quantified water parameters, corresponding to low-salinity and high-salinity waters. A total of 16,497 viral operational taxonomic units (vOTUs) were recovered, of which 85.59% were identified as novel viruses. β-diversity of viral communities supported the partition of samples based on salinity, and viral α-diversity differed significantly between low and high salinity. Taxonomically, Caudoviricetes dominated across all sites, with Myoviridae and Podoviridae more abundant in low salinity sites and Siphoviridae and Baculoviridae more abundant in high salinity sites. Gammaproteobacteria and Bacteroidota were the dominant host taxa, with distinct shifts in host abundance across the salinity gradient. Functional analysis revealed the abundant auxiliary metabolic genes involved in lipid, nucleotide, cofactor, and polysaccharide metabolisms. In particular, the alginate-degrading polysaccharide lyase family 6 was particularly abundant at high salinity sites. These results suggested that environmental factors, particularly salinity, shape the genomic diversity of estuarine viruses, which may further impact the biogeochemical processes in estuarine ecosystems.

**IMPORTANCE:**

Estuaries are highly dynamic ecosystems with strong environmental fluctuations, particularly in salinity and nutrients. This study highlights how environmental factors shape viral diversity and function by examining viral populations across the salinity gradient, providing new insights into viral dynamics in these ecosystems. We identified novel viruses and viral-encoded auxiliary metabolic genes in estuarine samples, including the discovery of a previously unreported viral alginate lyase gene that was abundant at high salinity sites, which sheds light on the ecological role of viruses in nutrient cycling and ecosystem partition. In addition, the study provides valuable information on distinct viral populations and virus-host interactions across the salinity gradient, which are essential for predicting ecosystem responses to salinity changes. These findings provide important implications for a broader understanding of microbial and viral ecology in estuarine ecosystems.

## INTRODUCTION

Viruses are essential players in global biogeochemistry because they control host abundance and metabolism (1). Viruses are abundant and widely distributed in natural ecosystems, including soil (2), glacial (3), marine, and freshwater ecosystems (4, 5). Marine and freshwater viral communities exhibit significant differences, as evidenced by extensive research conducted in oceanic and freshwater systems, including open oceans, coastal waters, lakes, rivers, ballast water (6–10), and sewage (11, 12). Viruses containing auxiliary metabolic genes (AMGs) were found in different ecosystems (3, 13), which might be important in mediating host metabolism and thus contribute to the global biogeochemical cycles. Initially, AMGs were discovered in the viruses infecting model organisms, such as photosynthesis genes found in cyanophages infecting *Prochlorococcus* (14). Since then, AMGs have been found across diverse metabolic functions, including carbon metabolism, lipid-fatty acid metabolism, motility, anti-oxidation, photosystem, sulfur, iron, copper metabolism, nitrogen cycling, DNA repair, and DNA replication initiation (15–22).

There are two widely adopted viral lifestyle strategies, the lytic and lysogenic lifestyles (23). The lytic lifestyle involves replication within host cells, followed by cell lysis to release progeny, whereas lysogenic viruses could integrate their genomes into host chromosomes and replicate passively (24, 25). It has been previously reported that aquatic viruses can interact with their hosts via Kill-the-Winner or Piggyback-the-Winner strategies (26, 27). These strategies may be dynamically regulated by different factors, including host abundance, environmental changes, and viral density (28, 29). Environmental factors could significantly affect life strategies and viral-host interactions, including salinity, temperature, light, and pH (30–33). Salinity, in particular, influences these interactions by altering prokaryotic communities and eukaryotic grazing, which ultimately affects the dynamics of viral infections (34).

Estuaries are intermediate zones where freshwater and seawater constantly mix, creating dynamic yet complex environmental conditions. These conditions impose substantial selective pressures on viruses and microorganisms, resulting in genetic diversification, metabolic adaptability, and community restructuring necessary for survival in such heterogeneous environments (35, 36). Recent increases in aquaculture activities, permanent human populations, and tourism have also brought profound ecological pressure to these estuaries (37). Therefore, in this study, three estuaries in Shenzhen, namely Pearl River Estuary (PRE), Shenzhen Bay (SB), and Dapeng Bay (DB), were selected to investigate the diversity and composition of DNA viruses. Specifically, this study aimed to (i) characterize the diversity, taxonomic composition, and host preferences of DNA viruses in estuaries of Shenzhen, with an emphasis on the impact of environmental factors, and (ii) identify novel AMGs within viral communities that have not been previously predicted in aquatic ecosystems. The results of this study provide new insights into the community structure of DNA viruses in estuarine ecosystems, focusing on how environmental factors influence viral diversity as well as the functional role of viruses in microbe-host interactions and nutrient cycling.

## MATERIALS AND METHODS

### Sample collection and physicochemical parameter analysis

Water samples were collected from three estuaries, including the PRE and estuaries adjacent to SB and DB (Fig. S1). Samples were collected primarily from four sites in the PRE (P5, P7, P8, and P9), two sites in the SB (P3 and P6), and four sites in the DB (P1, P2, P4, and P10). From each sampling point, 10 L of the surface water was collected for DNA viromic community analysis and physicochemical parameters quantification. Physicochemical parameters, including pH, salinity, total nitrogen (TN), total phosphorus (TP), conductivity, chemical oxygen demand (COD), nitrate (NO_3_^−^), nitrite (NO_2_^−^), ammonia (NH_4_^+^), and turbidity, were measured as described in our previous study (38). The *t*-distributed Stochastic Neighbor Embedding (*t*-SNE) was used to visualize high-dimensional data in a two-dimensional format using the R package Rtsne (version 0.16) for physicochemical parameters of 10 samples from the Shenzhen estuaries. The *t*-SNE parameters were configured as follows: theta = 0.5, maximum iteration = 1,000, and perplexity = 2.

### DNA extraction and viromic sequencing

Viruses were concentrated and purified from water samples according to the previously described procedure (39, 40). In brief, all samples were brought to the laboratory within 1 h and immediately pre-treated for concentration. First, 6 L of water from each sampling point was filtered through 800 mesh gauze, followed by secondary filtration with a 0.22 µm filter membrane. FeCl_3_-mediated flocculation was then used to enrich estuarine viruses, as previously described (4). Briefly, 6 mL of 1% aqueous FeCl_3_ was well mixed with 60 mL of the pooled estuarine water and was incubated at room temperature for at least 1 h to facilitate the formation of viral particle flocs. This FeCl_3_-treated water was passed through a polycarbonate 0.8 µm filter membrane (Millipore, Billerica, MA, USA). The 0.8 µm membrane was resuspended in 0.1 mol/L EDTA–0.2 mol/L MgCl_2_ buffer (pH 6.0), using 1 mL of buffer per 1 L of estuarine water (41), which was then mixed by rotation on a rotator at 4°C overnight, followed by centrifugation at 800 rpm for 5 min to collect the supernatant containing viruses. Viruses were further concentrated from the resulting supernatant using a 100 kDa Amicon Ultra-15 Centrifugal Unit (Millipore, USA). Following the manufacturer’s protocol, a 200 µL viral concentrate was used to extract DNA using the QIAamp MinElute Virus Spin Kit (Qiagen, Maryland, USA). DNA was quantified using a Qubit 4 Fluorometer (Thermo Fisher Scientific). Viral DNA was amplified by whole genome amplification with the Illustra Ready-To-GoTM GenomiPhi V3 DNA Amplification Kit (Cytiva, Marlborough, USA). Sequencing libraries were constructed using ALFA-SEQ DNA Library Prep Kits (FINDROP, Guangzhou, China) and sequenced on an Illumina NovaSeq 6000 platform using 2 × 150 bp paired-end chemistry at MAGIGENE Biotech Co., Ltd. (Guangzhou, China).

### Assembly and clustering of viral contigs

The viromic reads were quality-controlled using Trimmomatic v0.33 (42) with default parameters to remove sequencing adapters and low-quality sequences (3). SPAdes v3.15.2 was used to assemble both paired clean reads using the –meta –sc and –careful options (43). Viral prediction from assembled contigs using a combination of geNomad v1.7.0 (44), DeepMicroClass v1.0.3 (45), and seqkit2 v2.9.0 (46) (https://github.com/XiaomengWang0413/GDMCv). The specific analysis process was as follows: initially, geNomad was used to identify the viral sequences on the assembled sequences generated from the raw viromic assembly. Next, DeepMicroClass was applied to filter the results obtained by geNomad, removing sequences identified as non-viral by DeepMicroClass. Then, seqkit2 is used to screen the remaining sequences, retaining only those longer than 5 kb for subsequent analysis. Then, viral contigs were dereplicated at 95% identity with a minimum 85% overlap of the shorter contigs using CD-HIT v4.8.1 (47) to get the non-redundant 16,497 viral operational taxonomic units (vOTUs). The quality of these vOTUs was subsequently assessed using CheckV v1.0.1 48.

### Viral taxonomic assignment, lifestyle, and host predictions

To assign viral taxonomy for obtained vOTUs, three pipelines were used, including a viral genome homolog searching approach based on BLASTn alignment against the IMG/VR v4.0 database (49), a majority-rule approach based on BLASTp alignment against NCBI RefSeq viruses (9), and VPF-Class based on viral protein families (50). Identification of lytic and lysogenic lifestyles was performed according to the previously described (3). Briefly, lysogenic viruses were characterized using two recently developed approaches, including VIBRANT v1.2.1 (51) and BACPHLIP v0.9.3 (52). For BACPHLIP, a probability of 95% was applied. The iPHoP v1.2.0 (53) pipeline was used to predict phage–host associations, which has integrated tools including RaFAH v3.0 (54), WIsH (55), VHM (56), PHP (57), as well as BLASTn-based similarity search against host genomes and the CRISPR spacer database.

### Identification, annotation, and phylogenetic analysis of viral AMGs

Viral AMGs identification was performed following the previously described method (13). Briefly, AMGs were identified and annotated using the two pipelines: VIBRANT v1.2.1 using default parameters (51) and DRAMv v1.3.5 (58). Briefly, the VirSorter2 v2.2.3 (59) (--prep-for-dramv) was run on the vOTU sequences, and the AMGs (auxiliary scores < 4) were predicted from the resulting sequences by DRAMv. To investigate the evolutionary trajectories and structural conservation of the predicted viral polysaccharide lyase family 6 (PL6) gene, a BLASTp search was conducted against the UniProtKB database using predicted PL6 protein sequences from our viral contigs with an e-value threshold of 1e−10 to identify homologous sequences. The top 500 homologous sequences were retrieved. These sequences were aligned using MAFFT v7.520 (60), and the alignments were refined using trimAl (v1.4.1) (61) with the automated1 option. Trimmed alignments were input for phylogenetic tree construction via IQ-TREE v2.3.4 (−m MFP −bb 1000) (62). The phylogenetic trees were visualized and refined using the iTOL tool, enabling comprehensive evolutionary analysis (63). Phyre2 was used for structural similarity of the predicted viral PL6 protein (64). Phyre2 uses comparative modeling, relying on known protein structures to predict the target sequence’s structure, enabling the identification of reliable templates for modeling predictions of the conserved domains within the PL6 family. The quaternary structure was predicted using SWISS-MODEL, with a Global Model Quality Estimation score above 0.5 (65). The experimentally verified protein structure of bacterial PL6 and viral AMG protein structures were aligned and visualized using PyMOL (66), and the root mean square deviation (RMSD) was calculated.

### Novelty and abundance of vOTUs and AMGs

The vOTUs were compared to the IMG/VR v4.0 database (49), using BLASTn to assess the degree of novelty within the viral community. vOTUs were classified as known viruses if they had more than 90% nucleotide identity in at least 70% of the query or database sequence. Those with 50%–90% nucleotide identity in at least 70% of either sequence were categorized as similar viruses, and vOTUs that did not meet these thresholds were considered novel viruses (67). The index of vOTUs and viral genes was built using Bowtie2 v2.3.5.1 (68), and clean reads of each sample were mapped to the index of vOTUs and viral genes. Sorted BAM files generated using SAMtools v1.9 (69) were passed to CoverM v0.3.1 (https://github.com/wwood/CoverM) to filter low-quality mappings and calculate coverage across samples (parameters: -m rpkm tpm --min-read-percent-identity 95, --min-read-aligned-percent 0.90, --contig-end-exclusion 0, --no-zeros). Finally, the coverage value was denoted in the form of RPKM (reads per kilobase per million mapped reads) (70). The normalized relative abundance of the viral genes was calculated for each sample using the transcripts per million (TPM) method with CoverM v0.6.1 (https://github.com/wwood/CoverM) in contig mode (parameters: contig -t 24 --coupled -m TPM).

### Phylogenetic reconstruction, macro- and micro-diversity analysis of viral populations

The filtered reads from each sample were mapped to 16,497 vOTUs using Bowtie2 v2.3.5.1 (68). The generated BAM files, vOTUs, and read counts for each metagenome were then input into the MetaPop v1.0 (71), which performed pre-processing, macrodiversity, and microdiversity analysis. The MetaPop pipeline was run with the default settings (--snp_scale both), and genes from viral genomes were predicted using Prodigal v2.6 (72). Macrodiversity estimations include population diversity, including α-diversity and β-diversity. To correctly identify SNPs and estimate contig-level microdiversity, viral populations with average read depth coverage of >10× and genomic length coverage of >70% were retained for microdiversity analysis (71). Microdiversity was divided into local and global scales, with the local scale representing the set of variant loci identified independently within each sample and the global scale representing the set of variant loci identified over a genome across all samples. In order to evaluate the diversity of Caudovirales viruses in estuarine habitats, a phylogenetic tree was constructed using the terminase large subunit (TerL) protein sequence. The hmmsearch tool in HMMER v3.4 (73) was used to search the Pfam domain of the TerL protein of viral contigs against the Pfam 33.1 database (e-value threshold 1e-3) (74). The NCBI virus database (https://www.ncbi.nlm.nih.gov/labs/virus/, June 2024) retrieved viral reference TerL proteins. MUSCLE v5.1 (75) was used to align protein sequences containing the TerL domain, and then trimAl version 1.4.1 (-gappyout) was applied to trim the alignment (61). Using the evolutionary models chosen by IQ-TREE (-m LG + R4), phylogenetic reconstruction was carried out using the IQ-TREE v2.3.4 software (62). Finally, iTOL was used to alter the tree manually (63).

### Statistical analysis

Statistical comparisons of the α-diversity and environmental variables between samples were performed using the R package vegan (76) and ggplot2 (77). Bray-Curtis dissimilarity between samples was visualized using non-metric multidimensional scaling (NMDS) (metaMDS function in vegan). Similarity analysis (ANOSIM) was used to test the significance of differences in community structure and relative abundance between groups. The Mental test was used to show a correlation between physicochemical properties and viral communities, and the results were combined with the ggClusterNet package in R (78).

## RESULTS

### Grouping of estuary samples based on physicochemical factors

**Fig 1:**
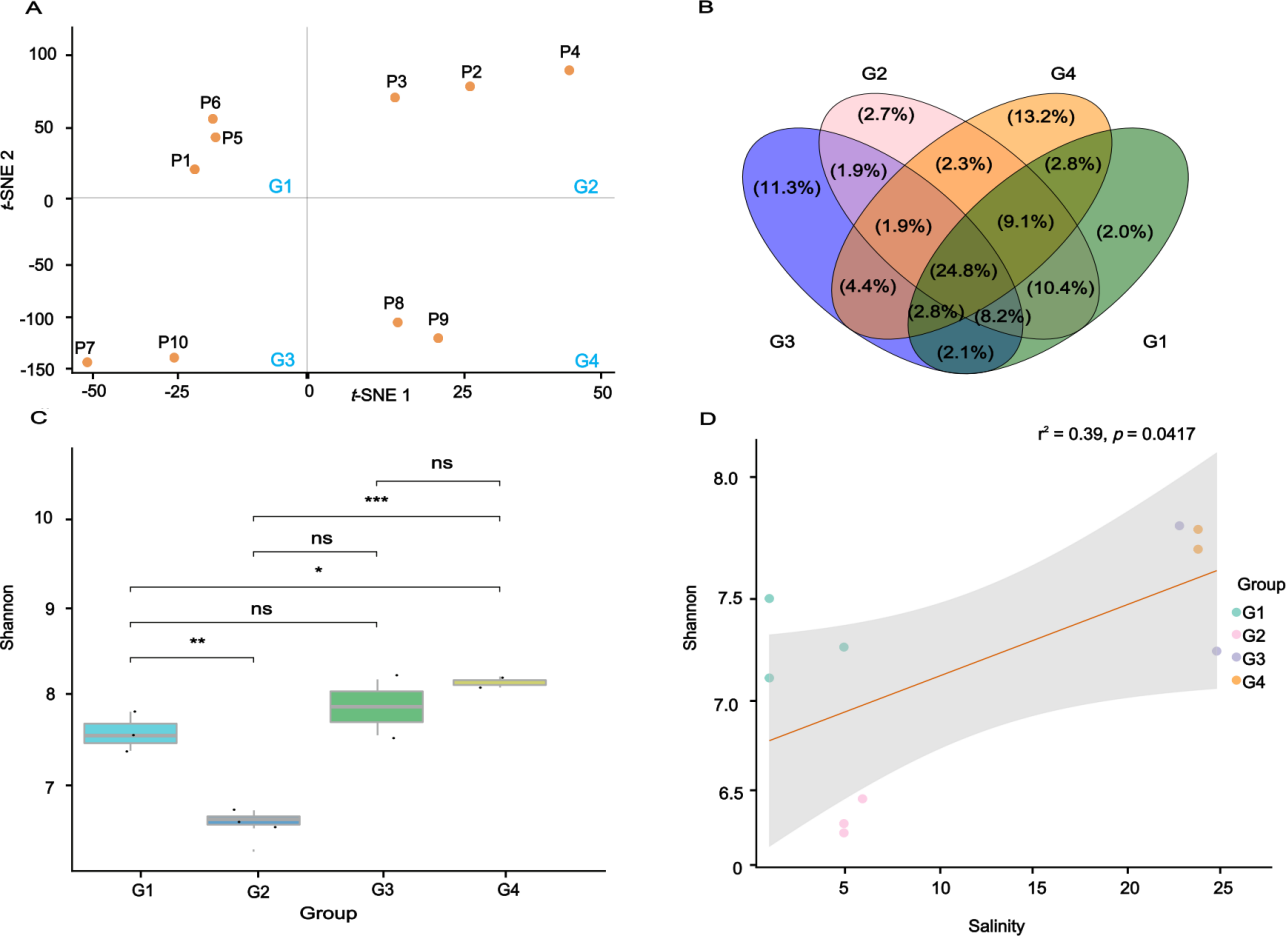
Grouping of samples, viral abundance, and diversity in estuaries. (**A**) *t*-Distribution stochastic neighbor embedding plot showing the assortment of samples based on water physicochemical indicators (tuning parameters used: theta = 0.5, maximum iteration = 1,000, and perplexity = 2). (**B**) Venn diagram showing the number of unique and shared vOTUs among estuarine samples. (**C**) α-diversity analysis of viruses between different groups (*t*-test, * indicating *P* < 0.05 significance, ** indicating *P* < 0.01 significance, and *** indicating *P* < 0.001 significance); NS, non-significant. (**D**) Relationship between salinity and vOTU α-diversity (Shannon index).

Physicochemical parameters, including pH, COD, TN, salinity, conductivity, nitrate, nitrite, ammonia, and turbidity, were visualized and classified using specific color scales to illustrate their variability across samples (Fig. S2). Samples were further categorized using *t*-SNE into four distinct groups: G1 (P1, P5, and P6), G2 (P2, P3, and P4), G3 (P7 and P10), and G4 (P8 and P9), with mean salinities of 1‰, 5‰, 25‰, and 23‰, respectively (Fig. 1A). Significant differences in salinity and conductivity were found between groups (Fig. S3). In particular, G3 and G4 had significantly higher conductivity and salinity values than G1 and G2 (*P* < 0.001), possibly due to local hydrodynamic conditions or anthropogenic influences. COD values were significantly higher in G2 and G4 (*P* = 0.002). Significant differences in nitrite concentrations were found between G3 and G1 (*P* = 0.003), G3 and G2 (*P* = 0.008), and G4 and G3 (*P* = 0.003), with G3 being the highest. These differences may reflect different nitrogen loads, such as agricultural or industrial effluent, in certain regions. Turbidity was significantly higher in G4 than in G3 (*P* = 0.039), probably due to suspended particles or pollutants in the water. Other parameters, such as TN, TP, ammonia, pH, and nitrate, showed no significant differences between groups.

### Overview of viromic sequencing analysis of estuarine DNA viruses

To evaluate the community composition and diversity of DNA viruses in different estuaries, 16,497 vOTUs were identified in estuarine samples, each represented by contigs ≥5 kb in size. Among them, 85.59% of vOTUs were classified as novel viruses, 11.56% as known viruses, and 2.85% showed similarities to previously identified viruses (Fig. S4). Meanwhile, 2.35% vOTUs were predicted to be potential proviruses based on CheckV, and the longest provirus was 201,620 bp in length (Table S1). The distribution of vOTUs revealed substantial overlap across groups, with the majority of common vOTUs found at the intersection of all four groups, sharing 24.74% of the total vOTUs (Fig. 1B). Despite the considerable overlap, each group exhibits many unique vOTUs, particularly G3 and G4, which have 11.32% and 13.19% unique vOTUs and exhibit apparent variation in the virus populations. In contrast, G1 and G2 share 10.35% of the total vOTUs. Furthermore, the overlap between G1 and G3 is comparatively minor, with only 2.10% of vOTUs shared between these groups. These results indicate that the higher salinity and conductivity in G3 and G4 likely drive both diversification and ecological specialization within viral communities. Taxonomically, 57.53% of 16,497 vOTUs were classified at the family level, and most of them were assigned to the class Caudoviricetes, while 42.47% were unclassified.

### Viral community diversity and environmental drivers in estuarine ecosystems

**Fig 2:**
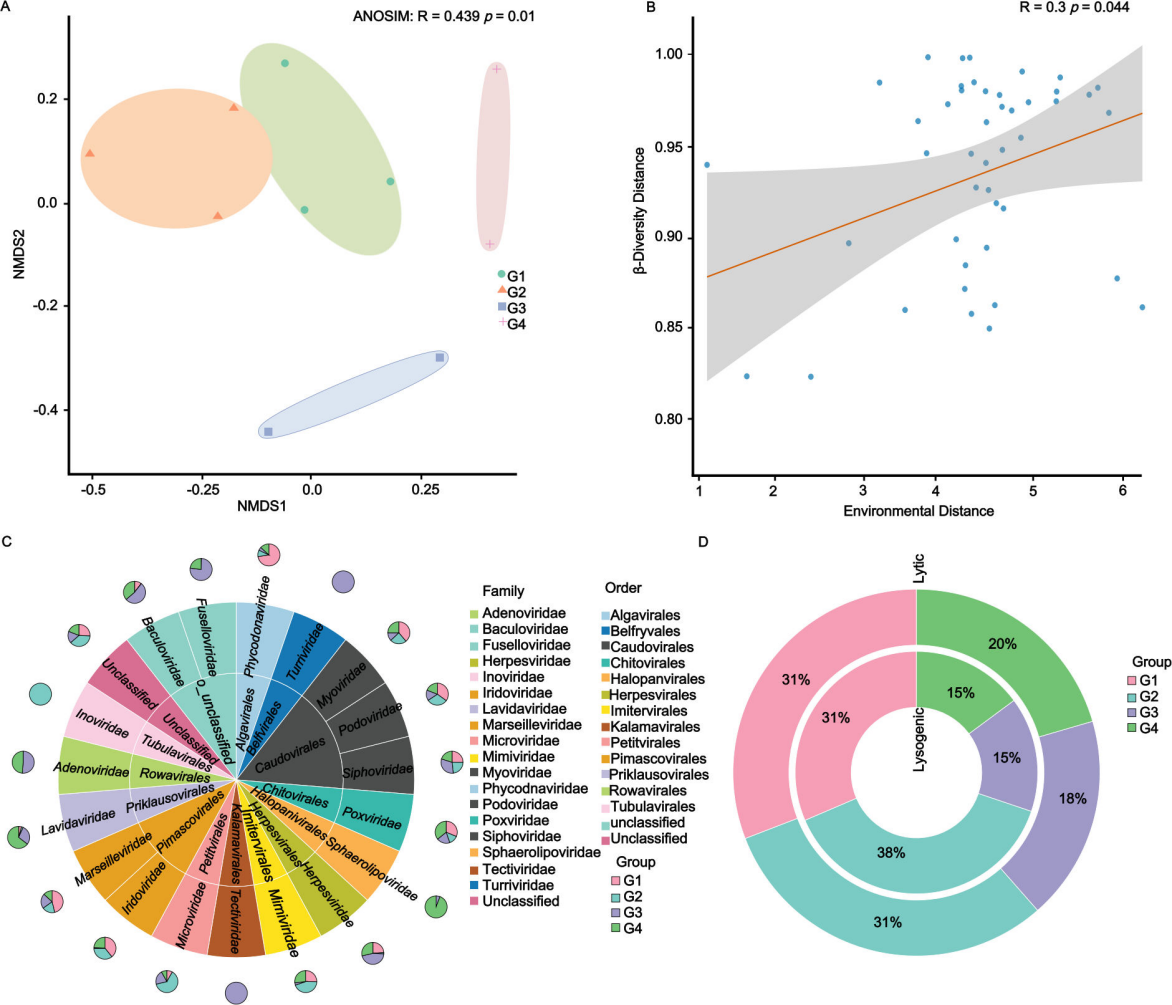
Diversity analysis of viral communities in the different estuaries. (**A**) NMDS analysis and ANOSIM of viral communities in estuaries. (**B**) The relationship between Bray-Curtis dissimilarity of vOTUs and environmental factors among different groups. (**C**) Comparison of viral taxa at the family level in different groups. (**D**) The relative proportion of predicted viral lifestyle.

The α-diversity analysis showed significant differences in viral communities across estuarine samples. The Shannon index was significantly higher in G4 compared to G2 and G1 (*P* < 0.001), reflecting greater viral diversity in high-salinity samples. However, no significant difference in diversity between G3 and G4 (Fig. 1C). Pearson correlation analysis showed a significant positive correlation between α-diversity and salinity (*P* = 0.0417) (Fig. 1D). β-diversity analysis (NMDS and ANOSIM) showed significant differences in community composition between estuaries (R = 0.439, *P* = 0.01), with G1 and G2 clustering closely and G3 and G4 clustering more distantly (Fig. 2A). Mantel correlation analysis of Bray-Curtis dissimilarity and all environmental factors showed a significant correlation (R = 0.3, *P* = 0.044) (Fig. 2B). Further analysis of individual environmental factors showed significant positive correlations with salinity (R = 0.356, *P* = 0.009) and conductivity (R = 0.359, *P* = 0.001), and a marginal correlation with pH (R = 0.214, *P* = 0.057). None of the other environmental factors were significantly correlated (Fig. S5). The viral composition across the four groups (G1, G2, G3, and G4) exhibited notable variation in the distribution of different viral families.

The taxonomically classified viromes in different groups were all dominated by the family Myoviridae (22.89%), Podoviridae (16.56%), and Siphoviridae (15.82%) (Fig. S6). Specifically, in G1, Myoviridae (28%), Podoviridae (19%), Siphoviridae (14%), and Phycodonaviridae (3%) were the most abundant viral families. The G2 exhibited a dominance of Myoviridae (17%), Podoviridae (16%), and Siphoviridae (12%), with a notable presence of unclassified viral families (54%). G3 was characterized by a high prevalence of Siphoviridae (29.3%) and Myoviridae (15%). In G4, Myoviridae, Podoviridae, and Siphoviridae were the dominant classified viral families. Unclassified viral families were abundant across all groups, with the highest presence in G2 (53.4%). Notably, Adenoviridae was absent in G1 and G2 but was present in G3 and G4, and Inoviridae was only detected in G2 (Fig. 2C). Some families, like Tectiviridae and Turriviridae, were particularly detected in G3. Overall, certain well-characterized families such as Iridoviridae, Myoviridae, Tectiviridae, Turriviridae, Inoviridae, and Lavidaviridae were dominant in specific groups. The relative abundance of putative lysogenic viruses was considerably lower than that of lytic viruses (6.2% and 94.8%) (Fig. S7). The relative proportion of lytic viruses was higher in G1 and G2 (31%) and lower in G3 (18%) (Fig. 2D). Conversely, the proportion of lysogenic viruses was higher in G2 (38%), followed by G1 (31%), while G3 and G4 exhibited the same proportion of lysogenic viruses (15% in both groups).

**Fig 3:**
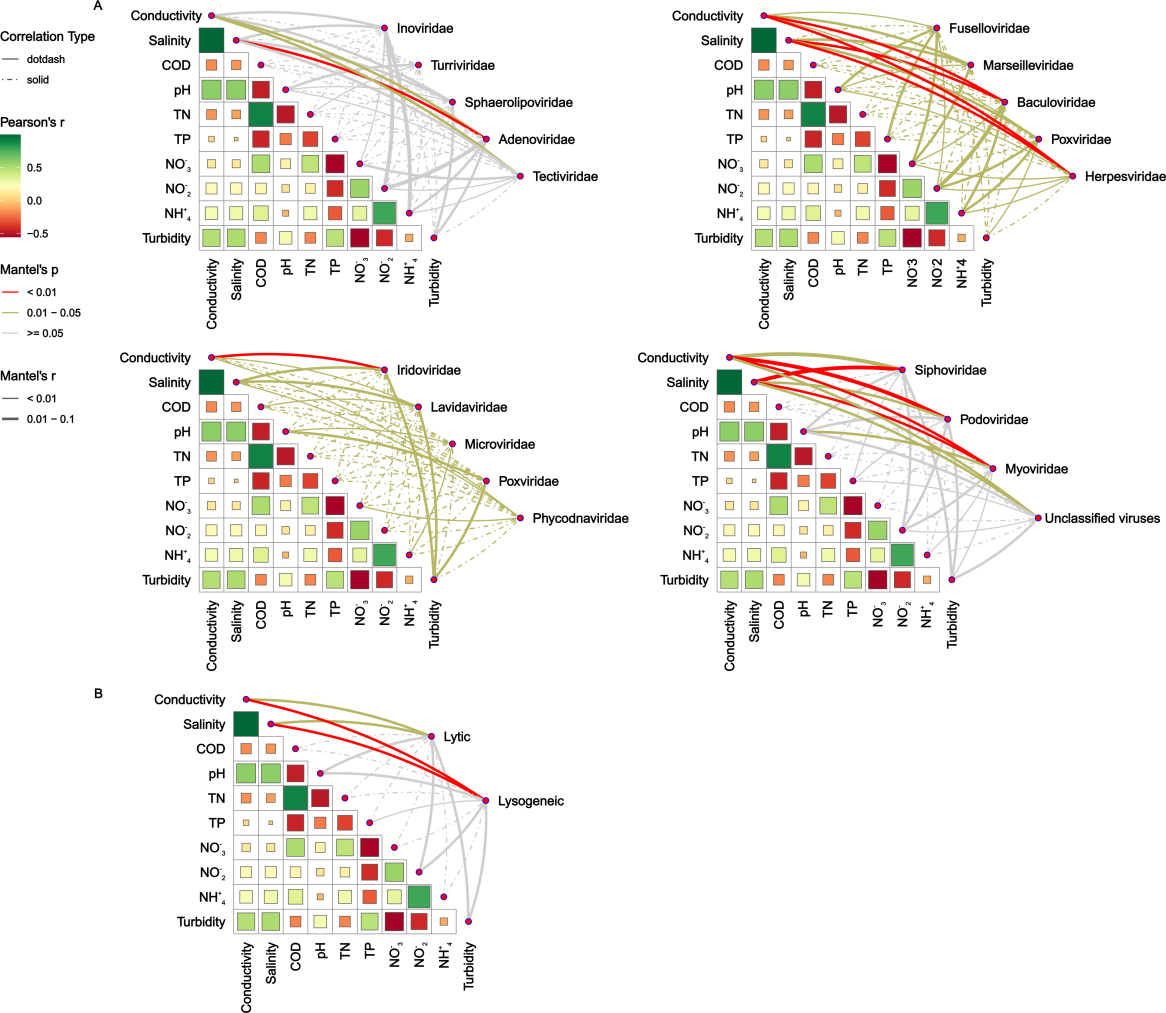
Environmental drivers of DNA viral community in the estuarine water samples evaluated by Mantel tests: viral families (**A**), viral lifestyle (**B**). Pairwise comparisons of water parameters are shown with a color gradient denoting Pearson’s correlation coefficient. The edge width represents the corresponding distance correlations (Mantel’s r). The line width corresponds to the Partial Mantel’s r statistic for the corresponding correlation, and the line color indicates statistical significance. Colors in red, green, and gray denoted the significant levels of *P* < 0.01, *P*: 0.01–0.05, and *P* > 0.05, respectively. Solid and dashed lines indicate positive and negative correlations, respectively.

Mantel correlation analysis was used to analyze the effects of abiotic factors on viral communities at the family level and viral life cycle. Mantel’s results showed that the taxonomic composition of dominant viral families Myoviridae, Podoviridae, and Siphoviridae was positively correlated with salinity, conductivity, and pH (Fig. 3A). For instance, the Phycodnaviridae was positively correlated with conductivity, salinity, and nitrate, while negatively correlated with pH, TP, and TN. About less abundant families, including Herpesviridae, Baculoviridae, Adenoviridae, and Iridoviridae, were significantly positively correlated with conductivity and salinity. The ssDNA family Microviridae was positively correlated with pH and ammonia. Lytic and lysogenic viruses correlate significantly with conductivity and salinity. However, lysogenic viruses exhibit a strong correlation with salinity and conductivity (*P* < 0.01) compared to lytic viruses (*P* = 0.01–0.05) (Fig. 3B).

### Host range and viral auxiliary metabolic functions

**Fig 4:**
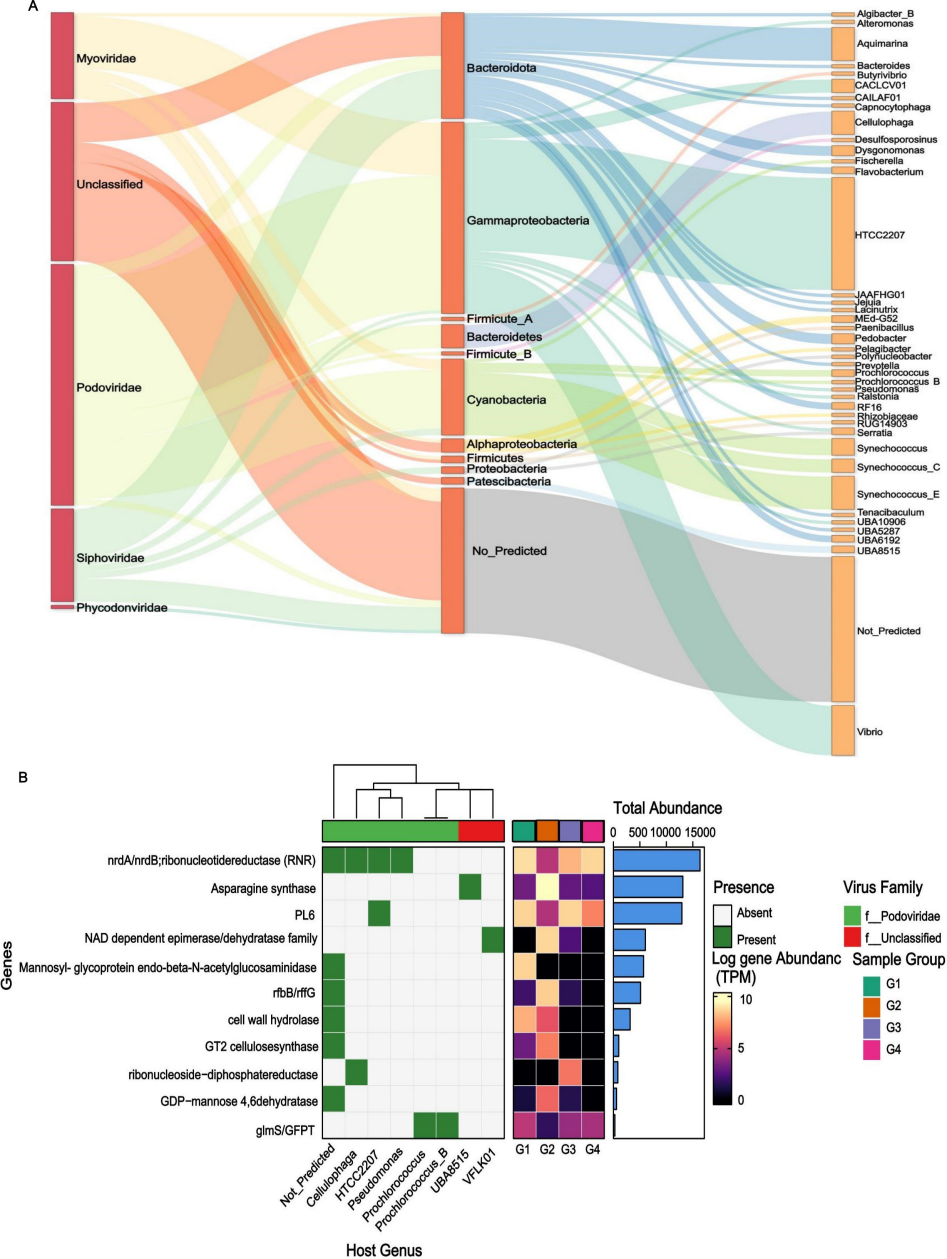
(**A**) Sankey diagram depicting the distribution of predicted virus-host linkages. The height of rectangles indicates the relative abundance of viruses at the family level and hosts at the phylum and genus levels (left to right). (**B**) The heatmap on the left displays the relative abundance (TPM) of detected viral AMGs in groups. The host phyla of the viruses carrying the detected AMGs are displayed on the right (green tiles). The color scale is shown as a log-transformed TPM.

Using iPHoP and VPF-Class, 9,872 out of 16,497 vOTUs were assigned to putative hosts. Most viruses were assigned to bacterial hosts, 167 vOTUs were assigned to archaeal hosts, and fewer were assigned to eukaryotic hosts. The dominant bacterial hosts were Proteobacteria (22.57%), Bacteroidota (15.39%), and Cyanobacteria (4.66%) (Fig. S8). Furthermore, the Sankey plot showed the association between viral families and putative viral hosts from phylum to class taxonomic ranks (Fig. S9). Proteobacteria had the highest prevalence of vOTUs (*n* = 3,567), followed by Bacteroidota (*n* = 1,754), Firmicutes (*n* = 862), Cyanobacteria (*n* = 581), Actinobacteriota (*n* = 436), Chloroflexota (*n* = 96), and Patescibacteria (*n* = 73). Archaea host phyla were mainly found in Thermoplasmatota (*n* = 85) and Thermoproteota (*n* = 26). Moreover, some viruses were capable of infecting eukaryotes (*n* = 23), linking them to hosts from two recognizable eukaryotic phyla (Gyrista and Chordata). Furthermore, we selected the top 100 most abundant vOTUs, and viral host interaction analysis indicated that Podoviridae was the most abundant family (41.5%), followed by unclassified viruses (27.3%). Siphoviridae (15.9%) and Myoviridae (14.8%) were also plentiful viral families. The diverse range of predicted host genera (41 genera) demonstrates the extensive host range of the abundant viruses. At the genus level, HTCC2207 (19.3%) and *Vibrio* (8.5%) were the most frequently predicted genera of class Gammaproteobacteria (Fig. 4A). The hosts were not predicted for the substantial proportion (25.0%) of viral contigs. The predominant AMGs were primarily involved in amino acid and carbohydrate metabolism, with additional genes associated with lipid metabolism, nucleotide metabolism, glycan biosynthesis, cofactor metabolism, and polysaccharide metabolism. Some AMGs appear site-specific, such as those encoding photosystem II, the phosphate transport system, and the information system, which were abundant in G3 and G4. In contrast, AMGs encoding the small subunit of ribosomal protein S21, peptidases, and methionine degradation were abundant in G1 (Fig. S10). Several AMGs were associated with specific hosts, such as pectate lyase in Bacteroidota and alginate lyase in Gammaproteobacteria and Bacteroidota. The AMGs in Patescibacteria were involved in cobalamin biosynthesis and information systems.

**Fig 5:**
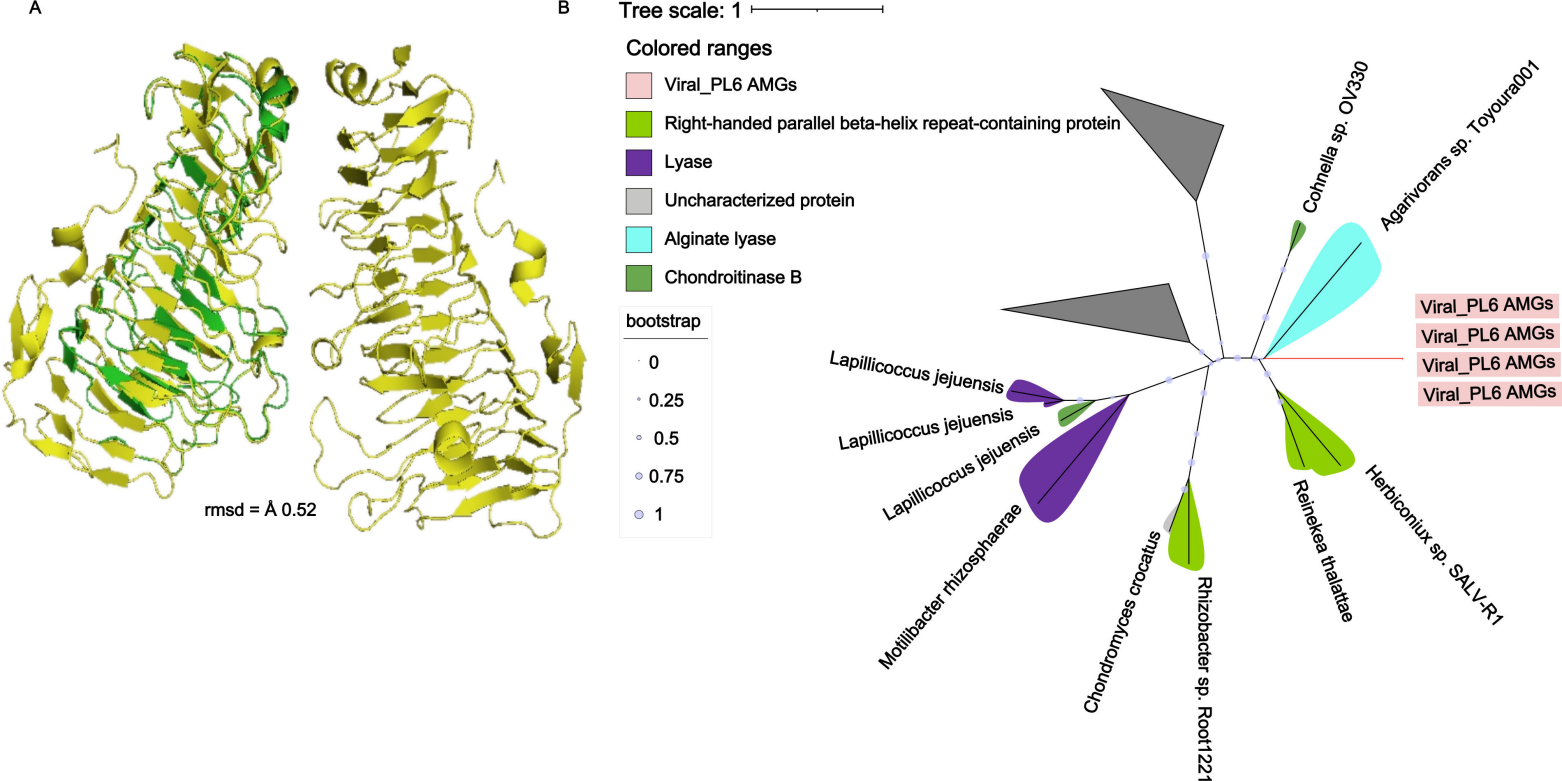
(**A**) Structural alignment of predicted protein structures was done using US-align to superimpose the models, and the diagrams were visualized in PyMOL, with α helices, β sheets, and loops depicted in green (predicted) and yellow (*Pseudomonas saltans* DSM 12145, PDB ID: 7O7A). (**B**) A phylogenetic tree of alginate lyase protein sequences, including four sequences from our viral AMG data set and 500 sequences retrieved from Uniprot via a BLAST search with default parameters, was aligned using Clustal Omega. The phylogenetic tree was constructed using IQ-TREE. Protein sequences from our study are indicated in pink in the tree, whereas retrieved protein sequences are colored in black.

Based on the 100 most abundant vOTUs, 11 viral AMGs were predicted to be involved in carbohydrate metabolism (PL6), nucleotide sugar biosynthesis (asparagine synthase, *glmS*/*GFPT*, and *rfbB*/*rfbG*), nucleotide metabolism (*nrdA*/*nrdB*), and starch and sucrose metabolism (GT2 cellulose synthase). Some AMGs, such as GT2 cellulose synthase and cell wall hydrolyase, were abundant in low-salinity samples, and *nrdA*/*nrdB* were abundant in high-salinity samples (Fig. 4B). In addition, we compared our identified AMGs with a global ocean AMG catalog and searched against databases. We identified that the PL6 AMG has not been previously described in viral genomes. A comparative structure analysis was performed to validate our findings further. The tertiary structure of the viral PL6 protein was validated by alignment with the bacterial PL6 experimentally validated protein structures. Structural alignments were performed using US-align and visualized in PyMOL, confirming their high structural conservation. The predicted structure was closely aligned with the alginate lyase from *Pseudopedobacter saltans* DSM 12145 (RMSD 0.52 Å) (Fig. 5A) and the alginate lyase from an Ignavibacteriales bacterium (RMSD 0.83 Å), indicating a nearly identical core architecture. Moderate similarity was observed with *Mucilaginibacter* sp. K268B poly-β-D-mannuronate lyase (RMSD 2.28 Å), while *Filimonas* sp. YR581 chondroitinase B-like protein (RMSD 3.48 Å) (Fig. S11). The phylogenetic tree demonstrated that the viral PL6 sequences clustered closely with *Agarivorans* sp. Toyoura001 (alginate lyase) (Fig. 5B), but were phylogenetically distant from the gammaproteobacterial host HTCC2207 alginate lyase (WP_039967638.1). These findings suggested that the viral PL6 genes were likely acquired through lateral gene transfer from a bacterium in the *Agarivorans* clade rather than acquired directly from the predicted host bacteria.

Moreover, viral host prediction suggests that many viruses are associated with Patescibacteria. Therefore, we extracted these viral contigs, performed phylogenetic analysis, and identified AMGs of these viruses. A maximum-likelihood phylogenetic tree of the TerL protein sequence was reconstructed using IQ-TREE, based on 786 global sequences from the IMG/VR database. The phylogenetic tree demonstrated that Patescibacteria-infecting viruses formed a cohesive clade, clustering with sequences from floodplains, groundwater, lakes, human oral microbiomes, rhizospheres, marine inlets, and non-marine saline/alkaline ecosystems (Fig. S12A). This broad environmental distribution suggests these viruses exploit conserved host interactions across disparate habitats. Functional profiling of viral genes identified 15 distinct AMGs in Patescibacteria-infecting viruses, with the RmlD substrate binding domain and NAD-dependent epimerase/dehydratase family being the most abundant, followed by the sulfotransferase family, particularly in high-salinity groups (Fig. S12B). Conversely, the 2OG-Fe(II) oxygenase family showed higher abundance in G1 and G2. The prevalence of RmlD, NAD-dependent epimerase/dehydratase, and sulfotransferase AMGs in high-salinity environments suggests metabolic adaptations that may enhance viral fitness in saline ecosystems and colonize ecological niches through metabolic reprogramming.

### Phylogenetic and microdiversity analysis of DNA viral communities

**Fig 6:**
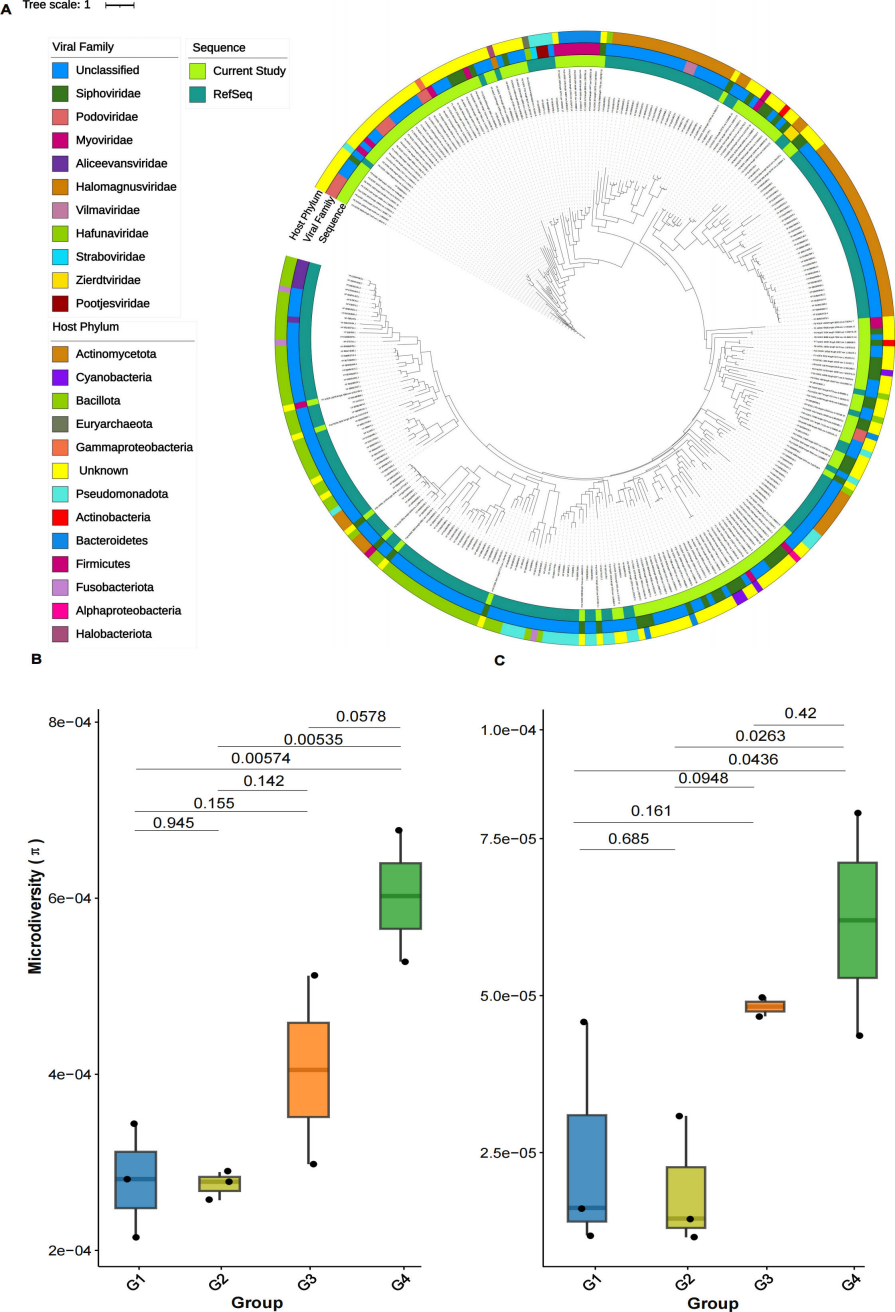
(**A**) Phylogenetic tree based on the TerL protein sequence of Caudovirales. Viral family-specific protein marker TerL was used to construct phylogenetic trees from estuarine viruses and viral RefSeq for Caudovirales. Viruses in RefSeq are colored in light blue, whereas estuarine viruses are indicated with red colors in the tree. The outer strip shows the host of estuarine and reference viruses. The microdiversity comparison of vOTUs at the local and global levels (B and C, respectively).

We used the TerL protein as a marker to assess the diversity and genetic distance of the estuarine virome. The phylogenetic tree was used to construct 175 unique vOTUs with the TerL protein sequence from estuarine samples and 216 from RefSeq. The topological structure indicated that most of the 62.65% vOTU sequences fell into unclassified DNA viruses. As shown in Fig. 6A, estuarine DNA viruses were widely distributed throughout the tree, with most of the sequences affiliated with Siphoviridae, followed by Myoviridae and Podoviridae. Although the Caudovirales are a well-characterized viral group, several sequences in the RefSeq database have not been identified as belonging to the Caudovirales. These clusters of previously uncharacterized diversity provided insights into the range of viral diversity. The viruses in the clades showed similar evolutionary patterns, and their phylogenetic distances further emphasized the diversity of Caudovirales in the current samples. Furthermore, we determined the microdiversity of vOTUs at local and global levels. The microdiversity of vOTUs increased from local to global scales, and the microdiversity of vOTUs at local and global scales was significantly higher in G4 and G3 while lower in G2 and G1 (Fig. 6B and C). This observation suggests that viral communities exhibit higher genetic variation and greater diversity at broader environmental scales. These results indicate that the estuarine virome harbors a rich diversity of viral populations, each with varying degrees of adaptation to the environment.

## DISCUSSION

There are several steep environmental gradients in estuaries, particularly salinity, which affect the diversity, functional activity, and distribution of viruses, prokaryotes, and eukaryotes (79, 80). Previous research has identified distinct differences in the composition and diversity of viruses in freshwater and marine ecosystems (81, 82). Our results showed that the salinity gradients significantly shape viral diversity and composition in estuarine ecosystems, consistent with previous studies that reported the different viral families exhibit distinct taxonomic and functional profiles depending on the salinity zone they inhabit (82, 83). Recently, studies on environmental viromes have uncovered numerous novel viruses in diverse ecosystems, including sediment (70, 84), ocean (9), freshwater (85), and soil (86, 87). The phylogenetic analysis in this study identified several new, previously unrecognized clades of DNA viruses. It highlights the enormous genetic diversity of largely uncharacterized viral populations in estuarine ecosystems. Despite the extensive research on viral taxa such as Caudovirales, a significant portion of DNA viruses remains unclassified, underscoring the substantial “viral dark matter” that persists across environmental systems (88). Future studies should focus on characterizing these novel clades, expanding reference databases, and exploring the ecological roles of these viruses in estuarine and other ecosystems.

Caudovirales were dominantly classified viruses, Myoviridae and Podoviridae, and Siphoviridae accounted for the most substantial proportion among estuarine samples. These results suggested that bacteriophages had numerical superiority in riverine ecosystems, consistent with the freshwater viromes in East Lake (12), Jiulong River (89), and Yangtze River in China (90). The nucleocytoplasmic large DNA viruses (NCLDVs) such as Phycodonaviridae, Iridoviridae, Mimiviridae, and Marseilleviridae were largely proportioned in low-salinity estuaries. This observation suggests that certain NCLDVs may be adapted to thrive in lower salinity environments, likely due to specific ecological and physiological factors that favor their replication in such conditions, and these findings are consistent with previous studies that examined the distribution of eukaryotic viruses, such as Circoviridae, Poxviridae, and Phycodnaviridae, in PRE (82). Phycodnaviridae and Mimiviridae are eukaryotic viruses infecting microalgae and protists, thereby contributing to biological carbon export from surface to deep layers in the aquatic ecosystems (91–93). Thus, many of these viruses in low salinity probably regulate carbon export and plankton populations in estuarine ecosystems (82). However, a large portion of the identified vOTUs in the estuarine virome remains unclassified, reflecting the limitations of existing databases in capturing the full spectrum of viral diversity. These limitations are the major obstacles to understanding the ecology and functions of viruses in environmental systems.

Viral-host interaction revealed notable salinity gradient signatures, with low-salinity environments favoring hosts such as Actinobacteria and Cyanobacteria, consistent with previous studies that members of Actinobacteria and Cyanobacteria-cyanophage interactions were dominant in freshwater ecosystems (94, 95). In contrast, the prevalence of Proteobacteria, Bacteroidota, Campylobacterota, and Firmicutes within predicted host taxa in high-salinity estuaries aligns with prior findings demonstrating their adaptations to osmotic gradients (96). These bacterial groups are frequently associated with nutrient cycling in estuarine and coastal ecosystems, particularly in organic matter degradation and nitrogen fixation (97). The high abundance of these predicted taxa suggests they may play key roles in sustaining biogeochemical cycles in these habitats, facilitating energy production through chemotrophic pathways (98). The detection of archaeal and eukaryotic hosts, albeit in lower abundance, suggests that viral communities in estuarine environments may affect a broader range of host taxa. The eukaryotic hosts, including Gyrista and Chordata, suggest potential viral effects beyond prokaryotic organisms. This broad host range, particularly the presence of archaea and eukaryotes, is consistent with co-evolution in virus-host interactions. A well-known ecological explanation for these observations is the Kill-the-Winner hypothesis (99). According to this model, when a host population becomes very abundant, it triggers a density-dependent signal that favors lytic phages infecting dominant hosts. This, in turn, leads to a sharp decline in the dominant host population, allowing less abundant hosts to thrive temporarily. Our results support this hypothesis, as the lytic life strategy of viruses was particularly dominant in low-salinity samples, where the abundance of certain hosts can fluctuate more rapidly. Further studies, particularly those focused on the interactions between phages and their host populations, are necessary to better understand the biogeochemical implications of viral predation in such sandwich environments.

Phylogenetic analysis of Patescibacteria-infecting phages indicates that they form a coherent cluster across various habitats, such as groundwater, the human oral microbiome, marine inlets, non-marine saline/alkaline ecosystems, and floodplains (Fig. S12). This suggests a stable evolutionary association between these viruses and their Patescibacteria hosts, regardless of environmental gradients. These results are consistent with a previous study reporting that groundwater viromes exhibit a consistent clustering of Patescibacteria-associated viruses across geochemical gradients, suggesting a stable host-phage connection (100). Contrary to the AMG repertoires of these phages demonstrating functional plasticity, characterized by niche-specific optimization of AMGs. For instance, the RmlD substrate binding domain and the NAD-dependent epimerase/dehydratase family are adapted for high salinity environments, while 2OG-Fe(II) oxygenases are prevalent in freshwater systems. These adaptations arise from local responses to environmental stressors (101), such as salinity and nutrient availability, rather than from phylogenetic divergence (102). Microdiversity (i.e., intra-population diversity) is reflected by nucleotide diversity, provides insights into selection pressures at the population level (71, 103), and can influence physiological traits (104) and ecological niches (105). Our study showed that viral microdiversity was lower in low salinity (G1, G2) and higher in high salinity environments (G3, G4). Viral microdiversity plays a significant role in viral quasispecies (106), and this phenomenon illustrates the intricate interplay between mutation, genetic drift, and selection, enabling viral populations to adapt to host defenses and environmental changes rapidly. The viral variants within a single population were lower in low salinity than in high salinity, suggesting that higher salinity enhances mutation rates and promotes more genetic variability, allowing a broader spectrum of viral variants to emerge, adapt, and maintain ecological resilience against environmental changes and host immune responses. These variations may reflect enhanced fitness and ecological resilience, enabling viruses to exploit a broader range of hosts and environments (103).

To date, the majority of viral AMGs have been identified in marine and terrestrial environments, and very little information is known about viral AMGs in estuarine ecosystems (87, 107, 108). Identifying AMGs in this study underscores the vital role of viral genes in mediating metabolic functions crucial for microbial survival and ecosystem processes. Specifically, AMGs related to amino acid, carbohydrate, lipid, and nucleotide metabolism were abundant, with notable variations across estuarine sites, highlighting their ecological importance in estuarine biogeochemistry. Interestingly, certain AMGs demonstrated site-specific distribution, with photosystem II and cobalamin biosynthesis genes prevalent in particular estuarine regions (G3 and G4), underscoring local adaptations among microbial communities. This distribution suggests that viruses in these areas may influence primary productivity by modulating photosynthesis and nutrient cycling, processes central to energy flow in the estuarine ecosystem (83). Other AMGs linked to encoding the small subunit of ribosomal protein S21, peptidases, and methionine degradation were commonly found in low-salinity settings, aligning with hypotheses that viruses selectively favor specific microbial functions vital to nutrient cycling (109).

**Fig 7:**
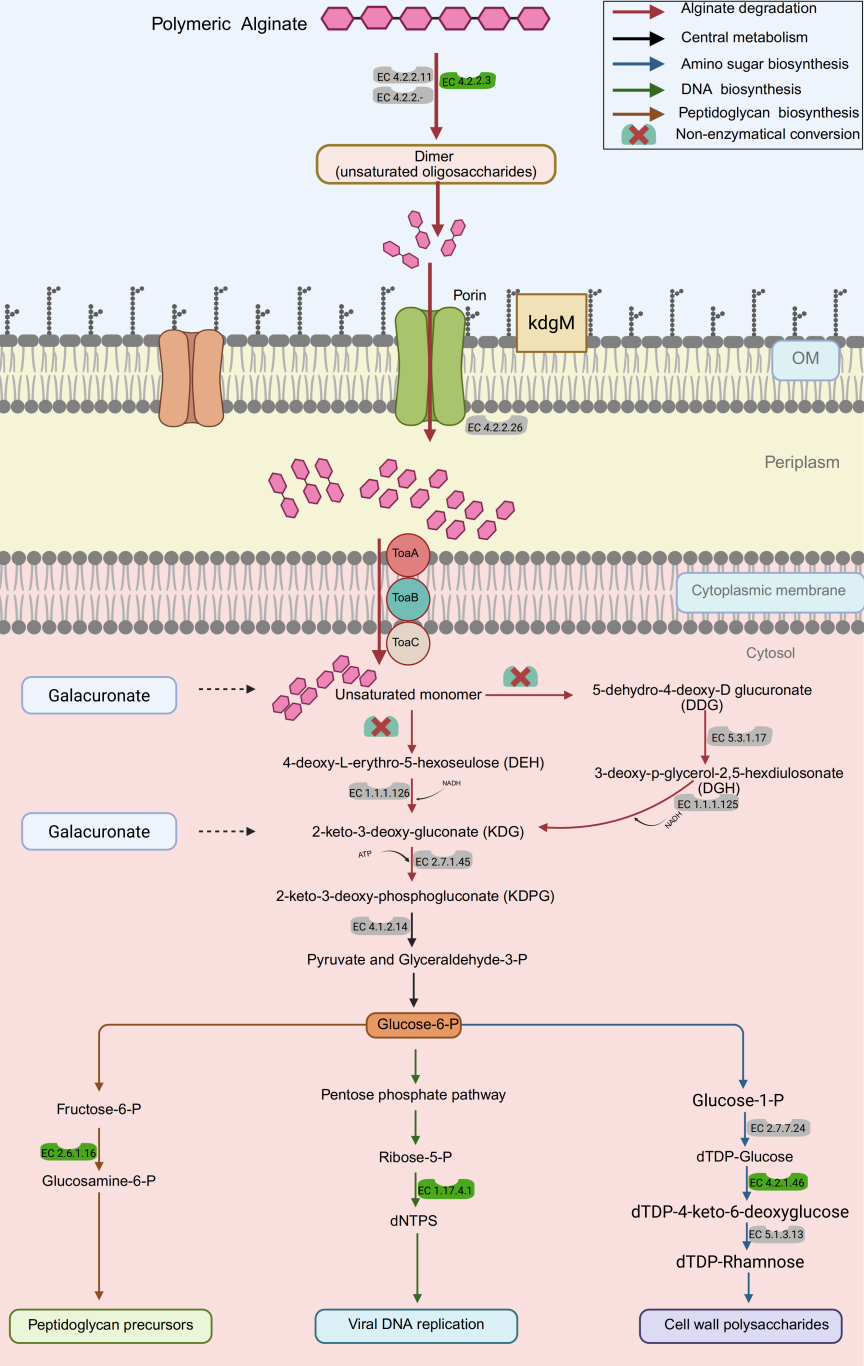
Constructed metabolic pathway of alginate degradation encoded by viral AMGs. EC numbers in green color indicate viral AMG identified in the current study, and EC numbers in gray color are bacterial enzymes reported previously (113, 114). OM, outer membrane; *kdgM*, oligogalacturonate-specific outer membrane porin; *toaABC*, oligoalginate symporter.

This work elucidates the alginate degradation process based on the discovered AMGs, as seen schematically in Fig. 7. In summary, alginate degradation occurs by the action of extracellular alginate lyase enzymes, which break down alginate into oligoalginates. These products are then translocated by specific transporters, including the outer membrane porin (KdgMN) and oligo transporters, facilitating the movement of alginate oligomers into the periplasm. Alginate lyases in the periplasm, including EC 4.2.2.26, further decompose unsaturated oligosaccharides into monosaccharides. Monosaccharides are transported to the cytoplasm via an inner membrane transporter, such as TaoABC, where host enzymes (EC 1.1.1.125, EC 2.7.1.45, EC 4.1.2.14) convert DEH, DDG, and DGH into 2-keto-3-deoxy-gluconate and 2-keto-3-deoxy-phosphogluconate, ultimately yielding pyruvate and glyceraldehyde-3-phosphate. Pyruvate enters central metabolism, while glyceraldehyde-3-phosphate participates in glycolysis and the pentose phosphate pathway (via glucose-6-phosphate), yielding ribose-5-phosphate and dNTPs (EC 1.7.4.1, AMG) essential for viral DNA replication. Glucose-6-phosphate is converted to fructose-6-phosphate, which aids in aminosugar biosynthesis (glucosamine-6-phosphate, EC 2.6.1.16, AMG) for peptidoglycan precursors. Additionally, glucose-1-phosphate is utilized for dTDP-rhamnose synthesis (EC 2.7.7.24, EC 4.2.1.46 [AMG], EC 5.1.3.13), contributing to cell wall polysaccharide production. These AMGs enhance viral metabolic functions by promoting alginate degradation. Alginate lyases encoded by viruses could play a critical role in the degradation of complex organic matter, enhancing organic carbon turnover and nutrient cycling within estuarine ecosystems (110). Estuarine environments, with their fluctuating salinity and nutrient dynamics, likely exert strong selective pressures on viral populations, favoring the acquisition of AMGs that enhance host metabolism and ecosystem functioning (111). These AMGs and their site-specific distribution suggest that viral genomes are highly dynamic and rapidly evolving in response to changing environmental conditions and the need to adapt to different host organisms. This aligns with the Red Queen hypothesis (112), which posits that continuous co-evolution between hosts and pathogens drives adaptive genetic changes in both parties.

### Conclusion

This study highlights the intricate complexity of viral diversity and ecological roles in estuarine environments, where salinity gradients profoundly shape viral community composition and functionality. Taxonomic and phylogenetic analyses identified distinct viral clades, primarily within the Caudovirales, corroborating observations from other riverine viromes. However, the widespread presence of unclassified viral sequences highlights the concept of viral dark matter and reveals significant gaps in our understanding of viral diversity in estuarine ecosystems. Host-virus interactions were closely linked to salinity, with Actinobacteria and Cyanobacteria serving as primary viral hosts in low salinity zones, and Proteobacteria and Firmicutes dominating in high salinity zones. The AMGs in the viral genomes underscore the important role of viruses in host metabolic processes, particularly in amino acid, carbohydrate, and lipid metabolism. In addition, the novel discovery of an alginate lyase gene highlights the adaptive evolution of viral genomes in response to organic nutrients, highlighting their potential contribution to carbon turnover in estuarine ecosystems. Overall, this research highlights the complex interactions between viruses, microbial communities, and their environment. Further investigations are required to classify uncharacterized viral clades, clarify virus-host interactions, and improve our understanding of the broader ecological role of viruses in estuaries and other ecosystems.

## Data Availability

The sequencing data have been deposited in the NCBI database under BioProject no. PRJNA1214496.

## SUPPLEMENTAL MATERIAL

Supplemental figures aem.00407-25-s0001.docx Figures S1 to S12.

Table S1 aem.00407-25-s0002.xlsx Quality metrics of estuarine virome near the Shenzhen coast.

ASM does not own the copyrights to Supplemental Material that may be linked to, or accessed through, an article. The authors have granted ASM a non-exclusive, world-wide license to publish the Supplemental Material files. Please contact the corresponding author directly for reuse.
