# Supplementary material for: Salinity-driven shifts in estuarine viral community composition and diversity near the Shenzhen coast
Source: Appl Environ Microbiol. 2025 Jul 2;91(7):e00407-25. doi: 10.1128/aem.00407-25 (PMC12285232; doi:10.1128/aem.00407-25)
Supplement: Supplemental figures — Figures S1 to S12. [file aem.00407-25-s0001.docx]

**
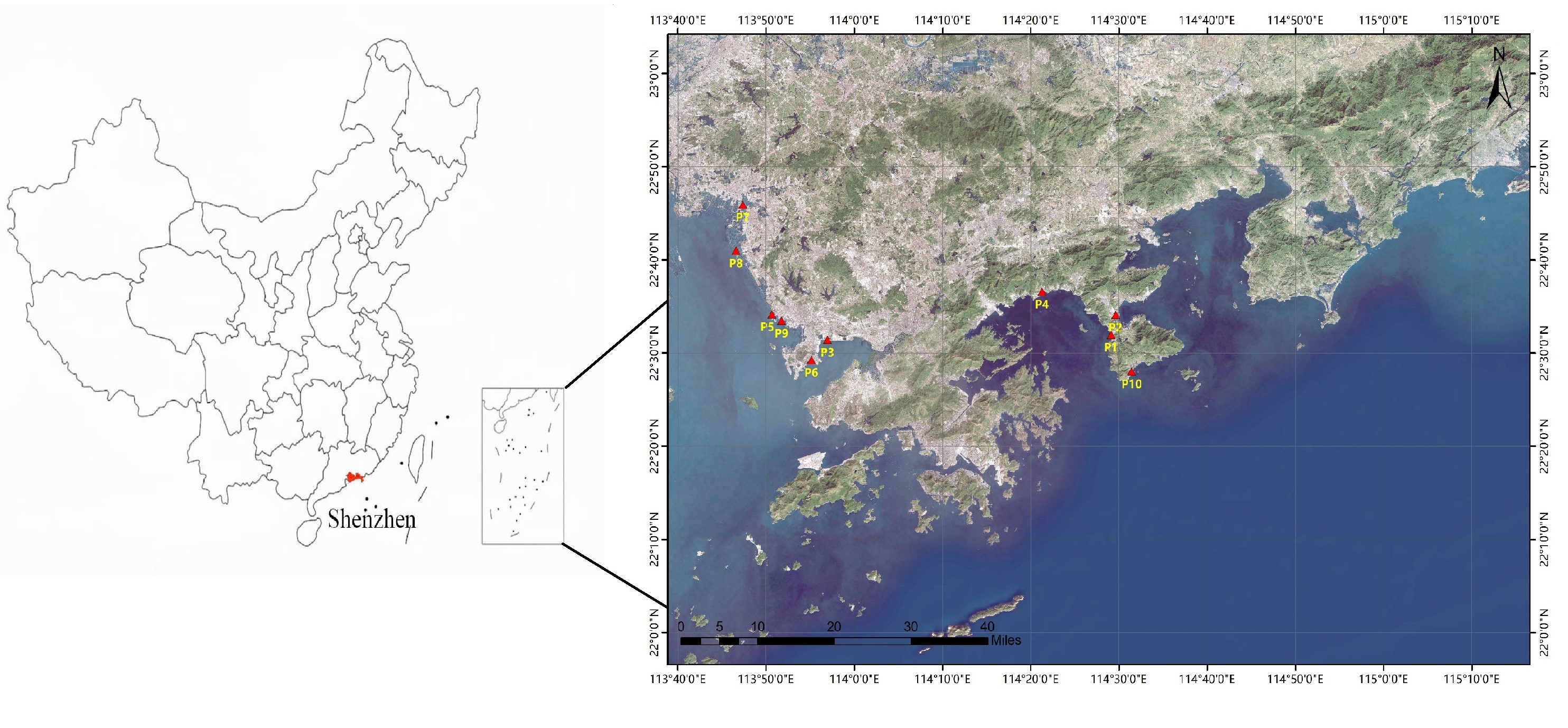
Figure S1**. Map of the Shenzhen coastal area with sampling sites. Red dots indicate the sampling sites.


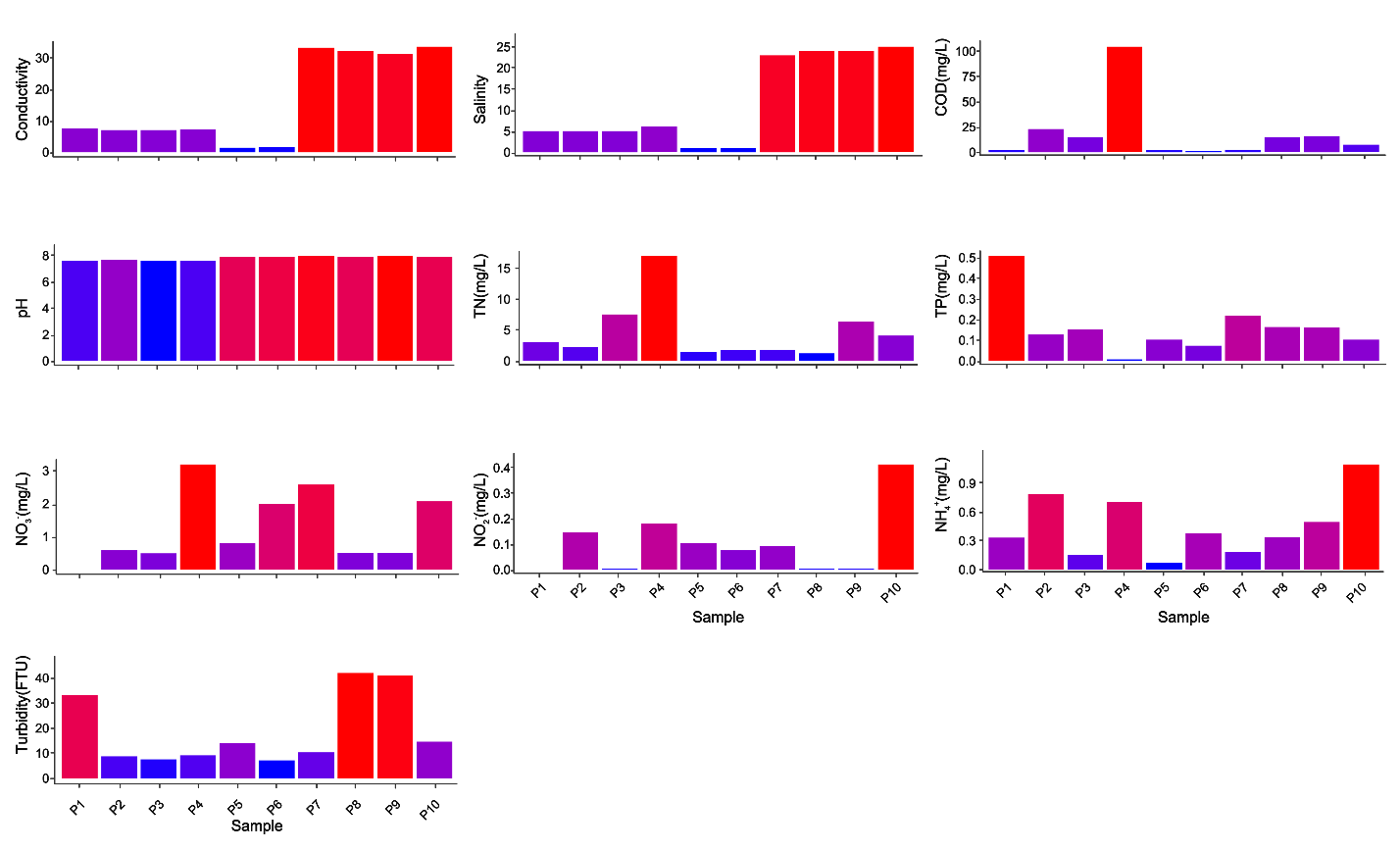
**Figure S2.** Visualization of water parameters across different sites. Each parameter is color-coded using specific scales to reflect variations in its physicochemical composition. The color gradients represent the range of values observed for each parameter.


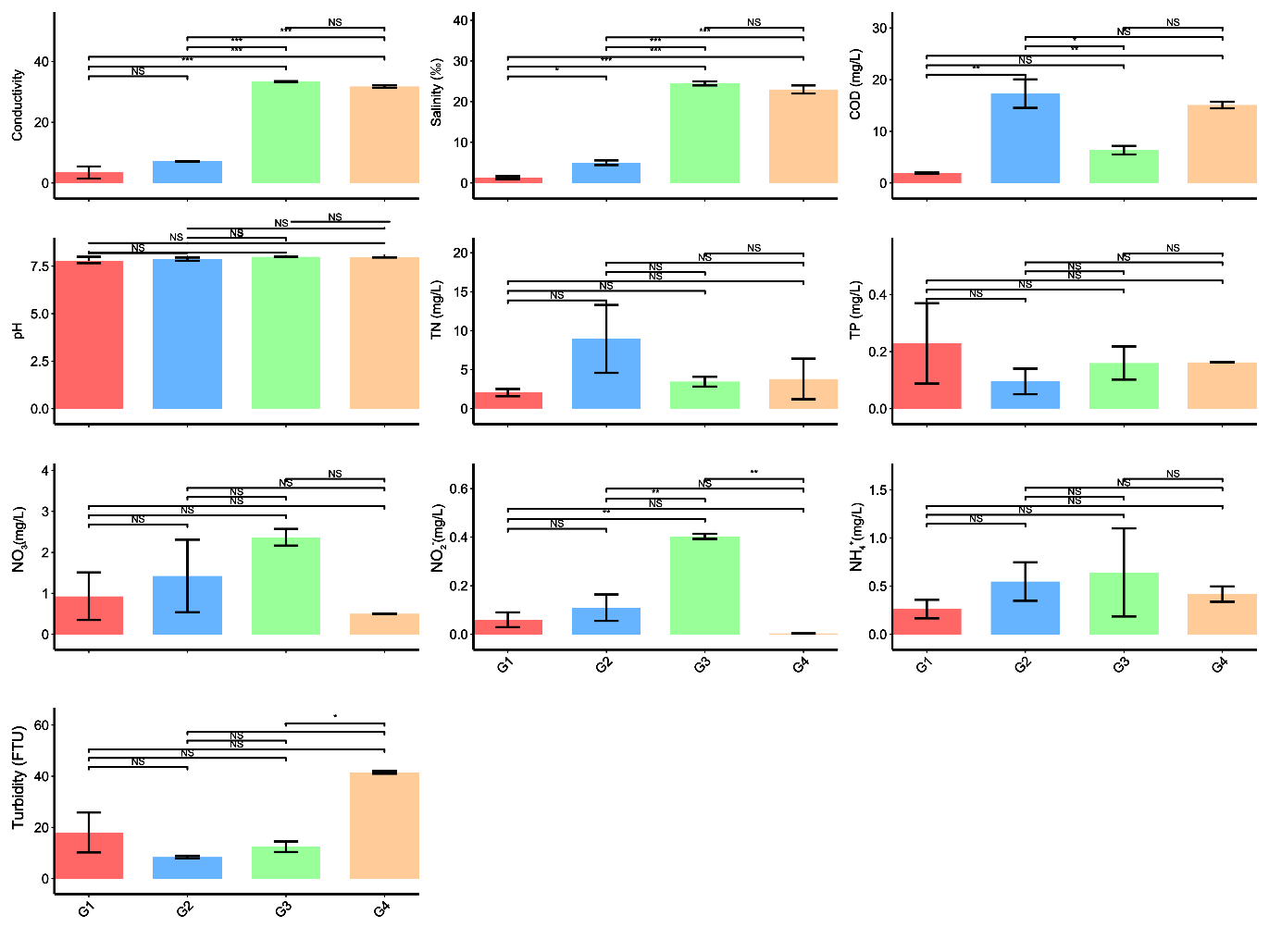
**Figure S3.** Water physicochemical parameters comparison between different groups (T-test, * indicating significance, *p* < 0.05, ** indicating significance, *p* < 0.01, and *** indicating significance, *p* < 0.001), NS. Non-Significant.


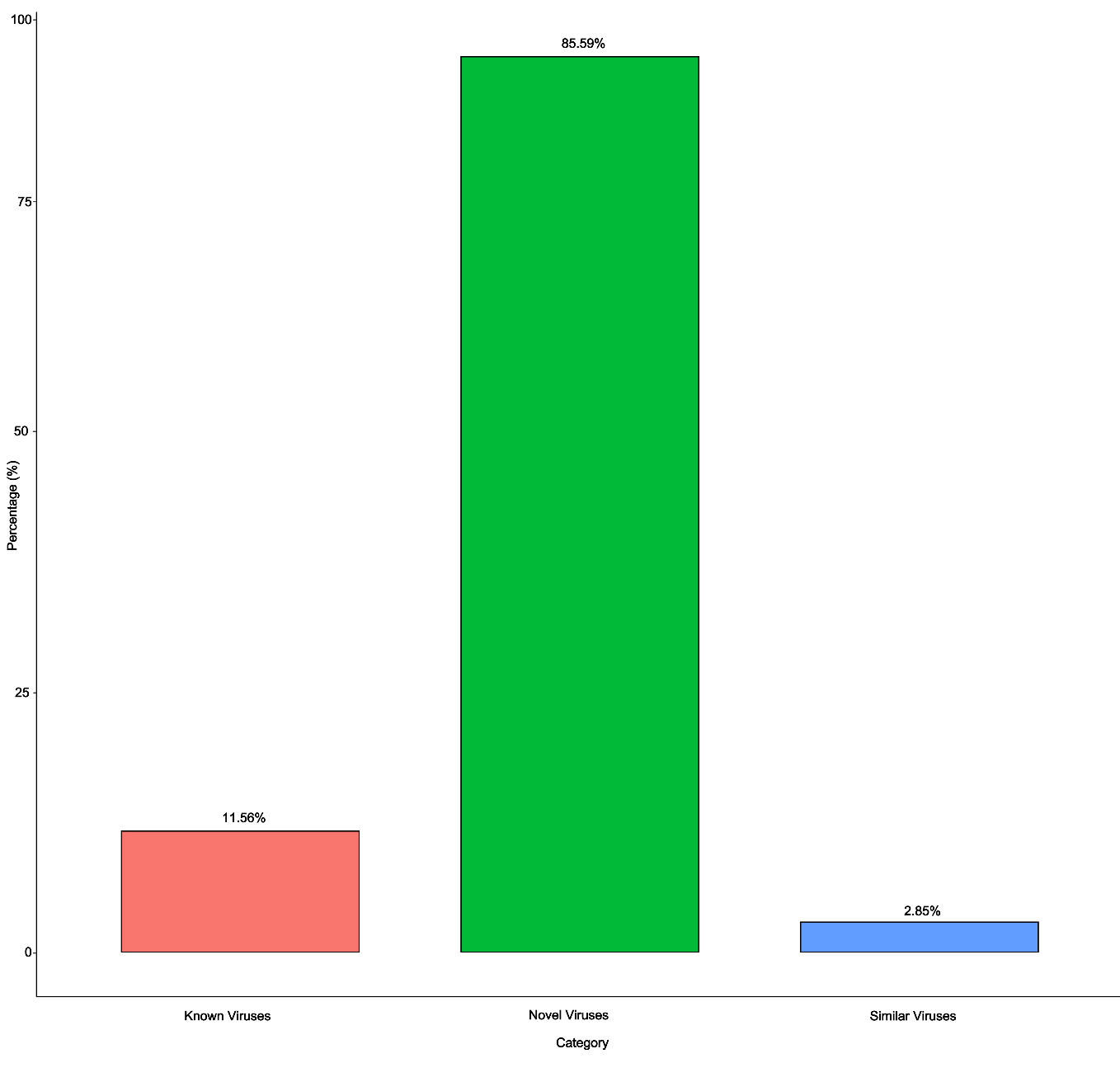
 **Figure S4**. Estimation of viral novelty based on vOTU nucleotide sequence homology with the IMG/VR v4 database.


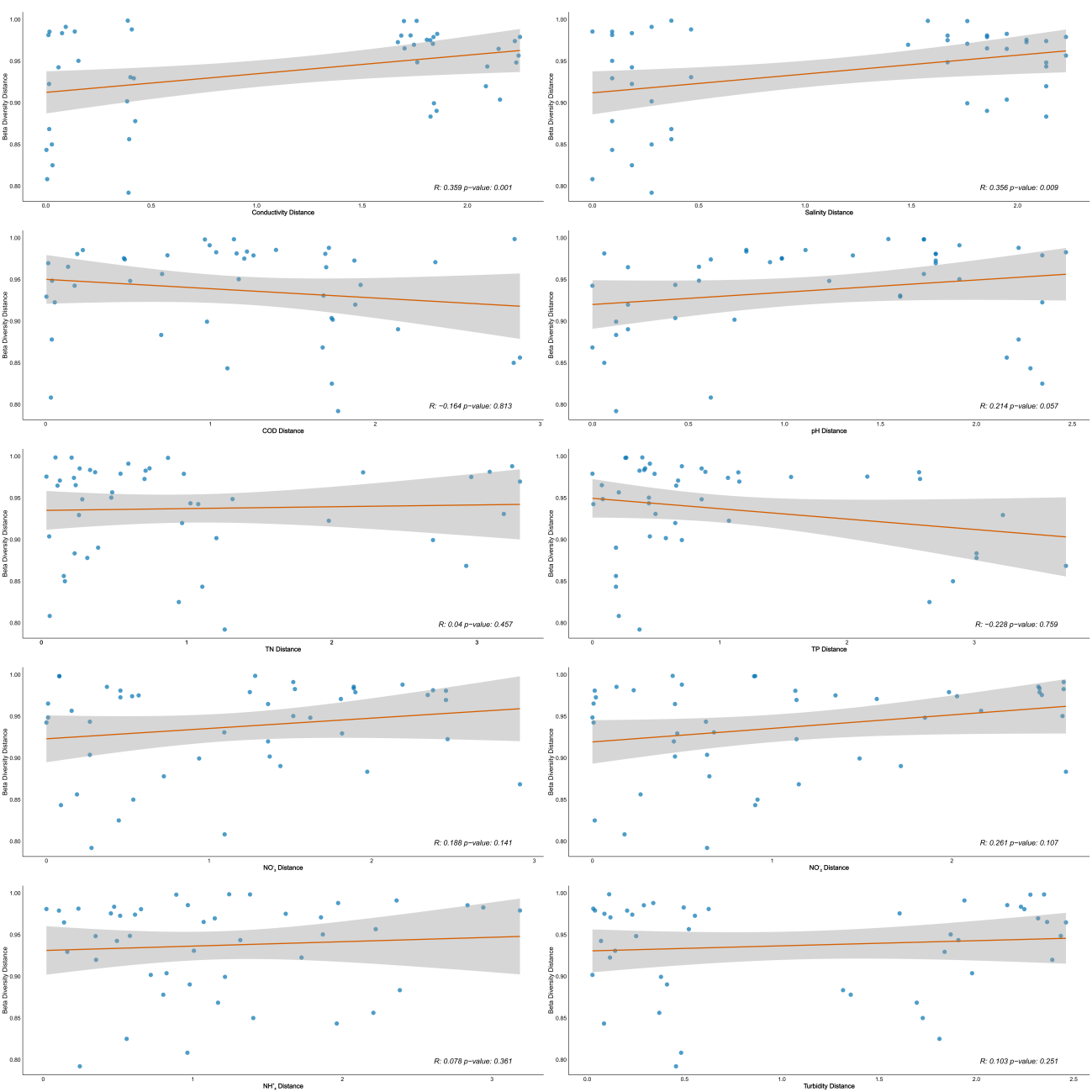


**Figure S5.** Relationship between Bray-Curtis dissimilarities and individual environmental factors.


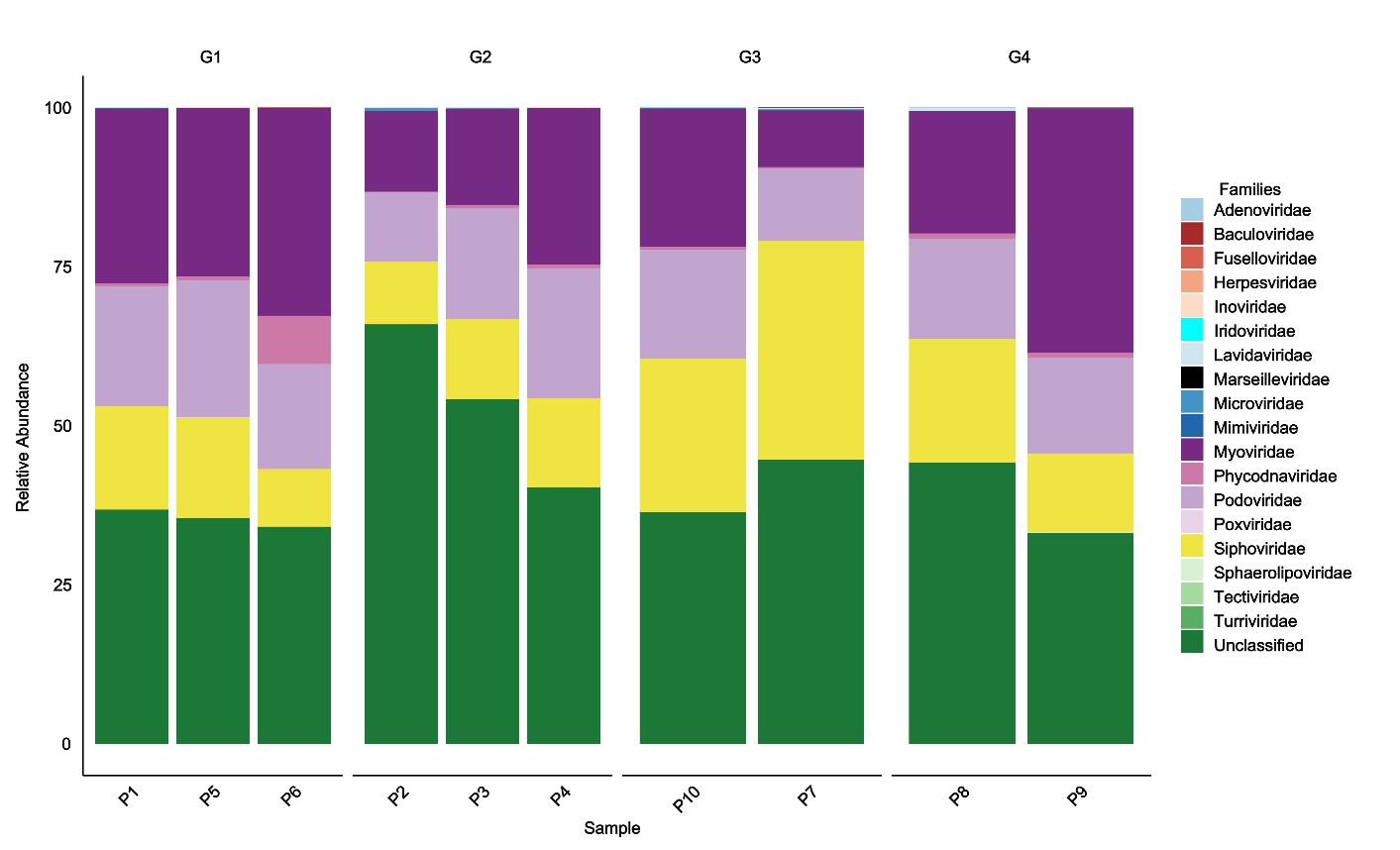


**Figure S6**. The relative abundance of the virus family-level classification for samples from different groups


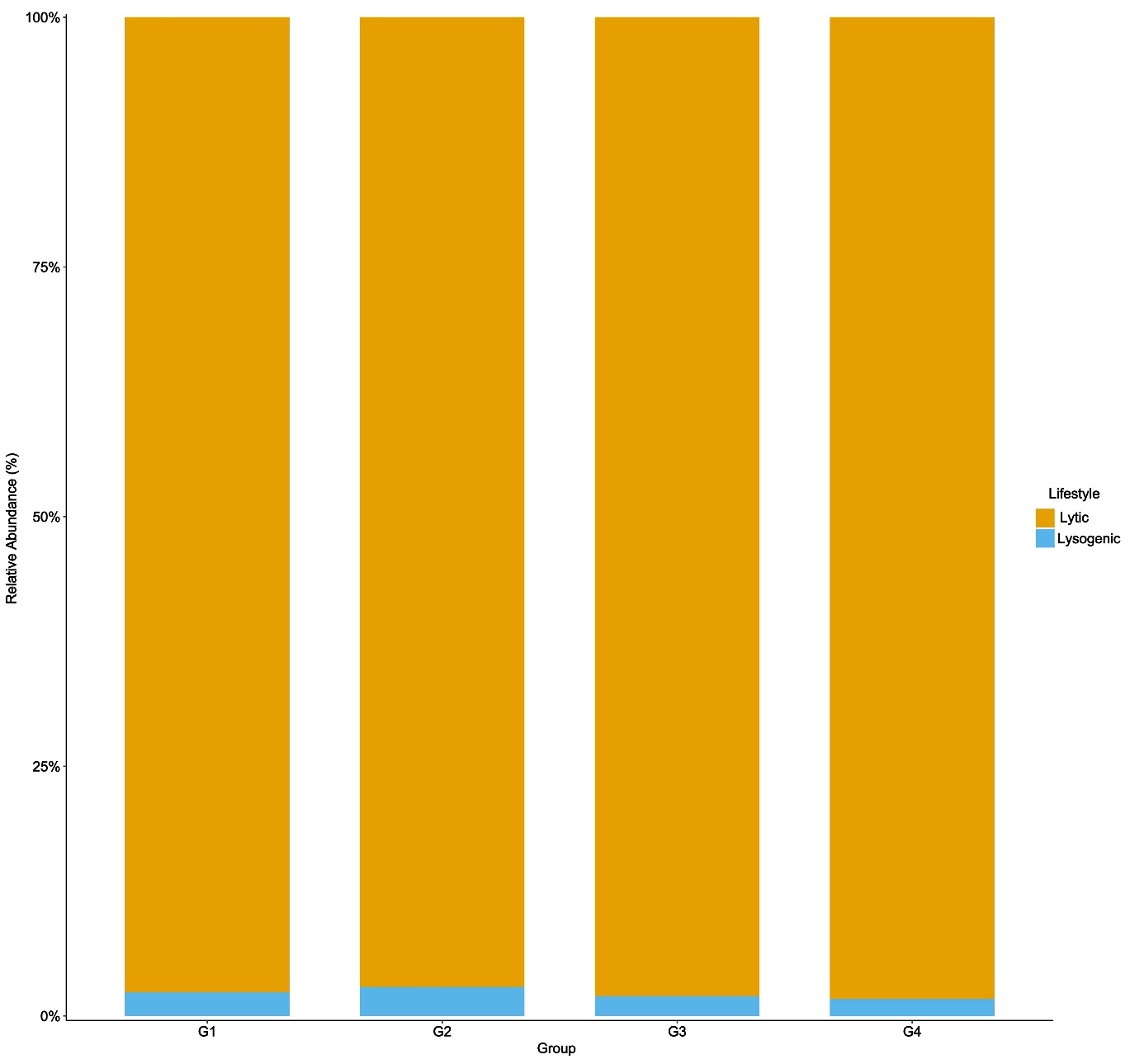


**Figure S7**. Relative abundance of predicted viral lifestyles in different groups.

**
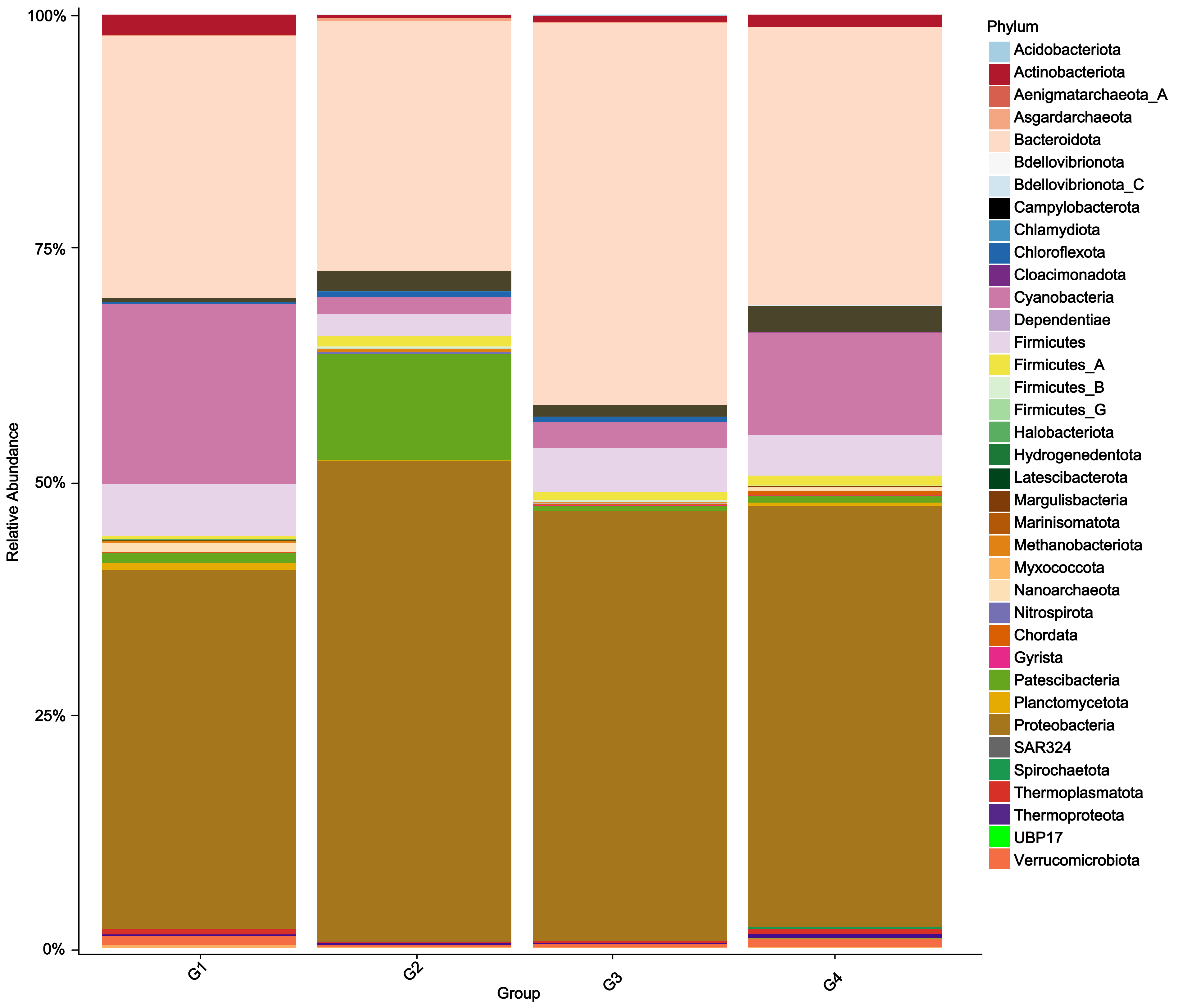
Figure S8**. Summarized viral abundances based on their host phyla. The relative abundance was calculated based on the reads mapped per kilobase of contig per million mapped reads (RPKM) across each sample.


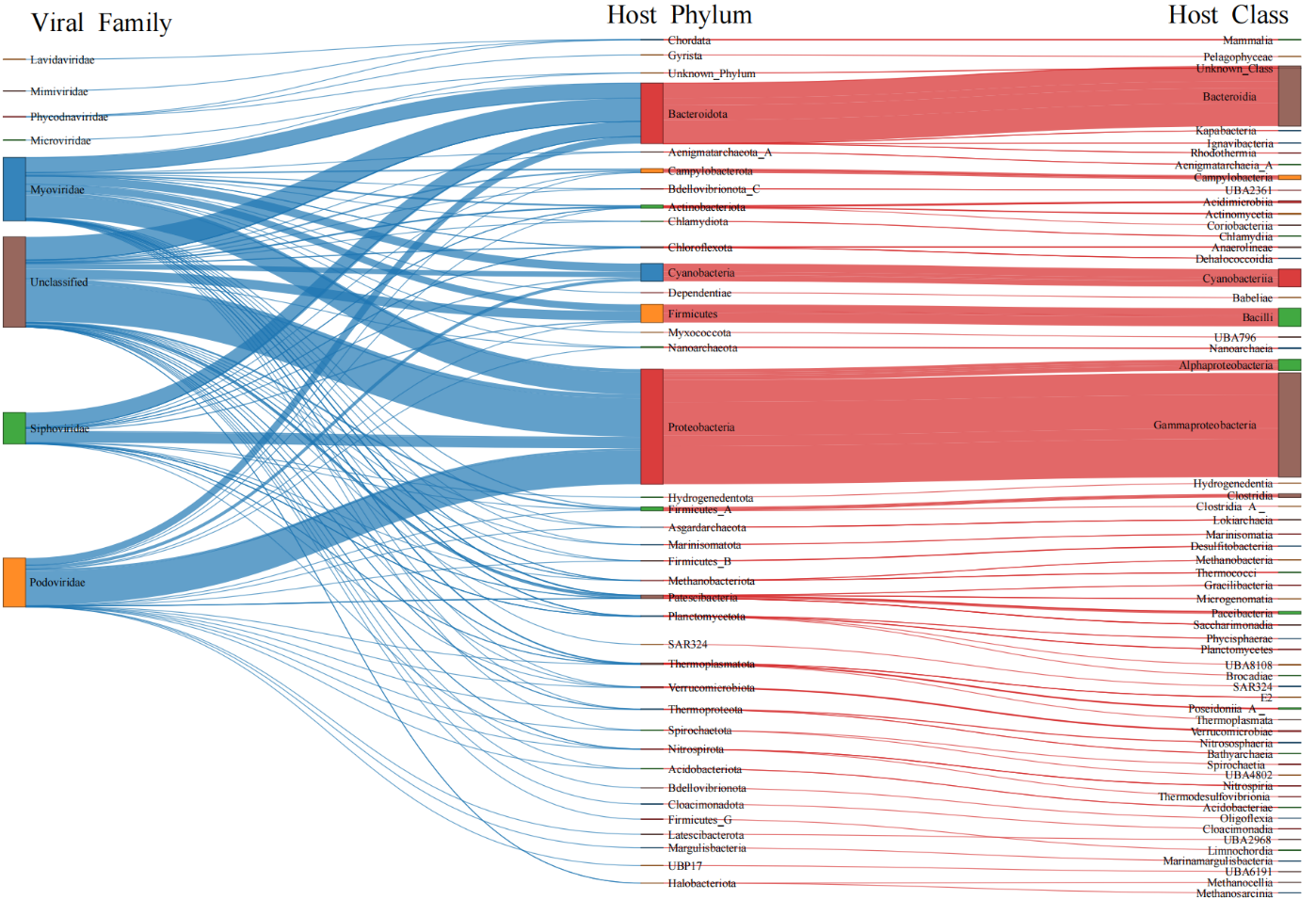


**Figure S9**. Sanky diagram depicting the distribution of predicted virus-host linkages. The height of rectangles indicates the relative abundance of viruses at the family level and host at phylum to class level (Left to Right).


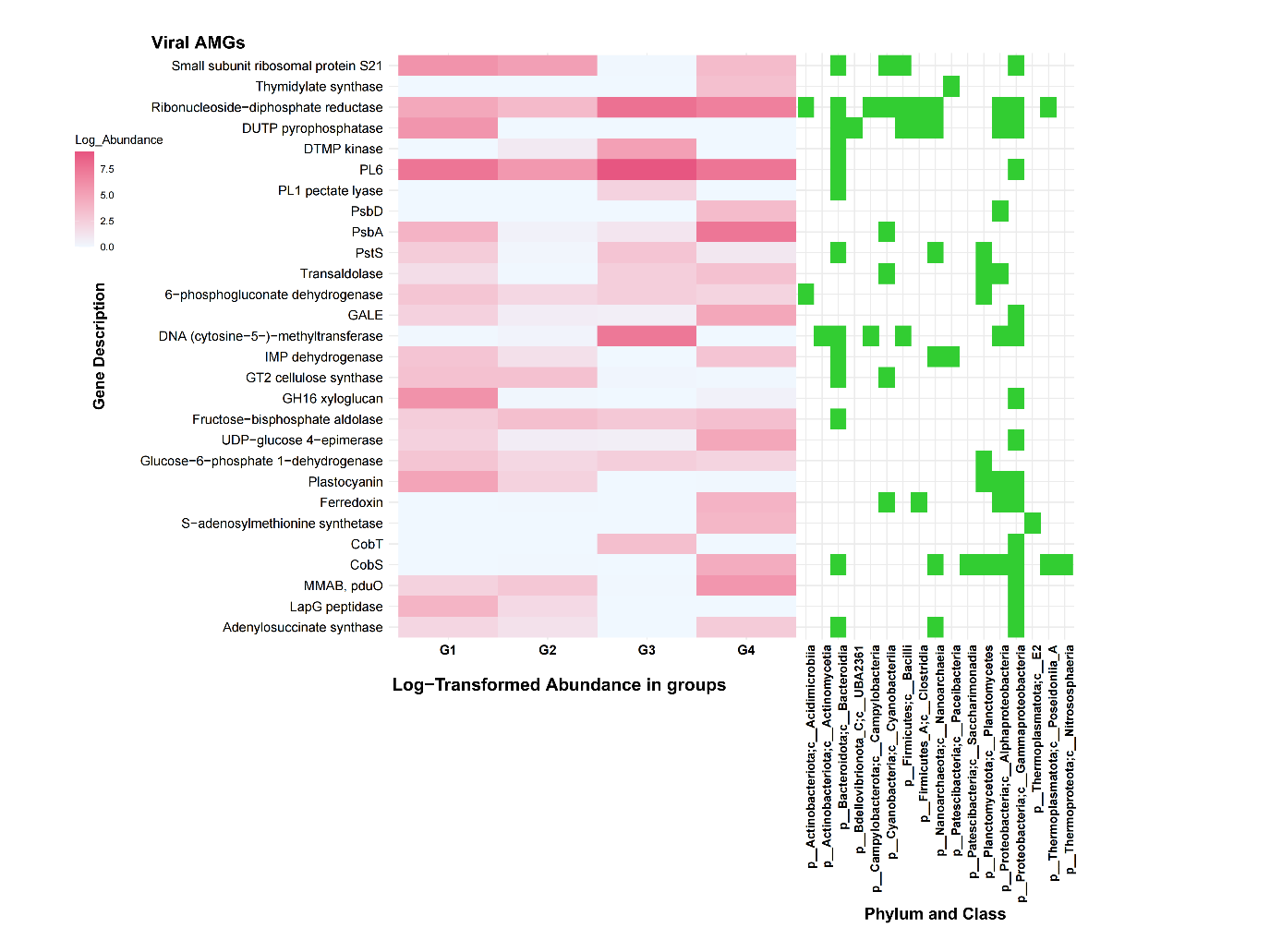


**Figure S10**. The heat map on the left displays the relative abundance (TPM) of predicted viral auxiliary metabolic genes (AMGs) within each group. The host phyla of the viruses carrying the predicted AMGs are displayed on the right (green tiles). The color scale is shown as a log-transformed (TPM).


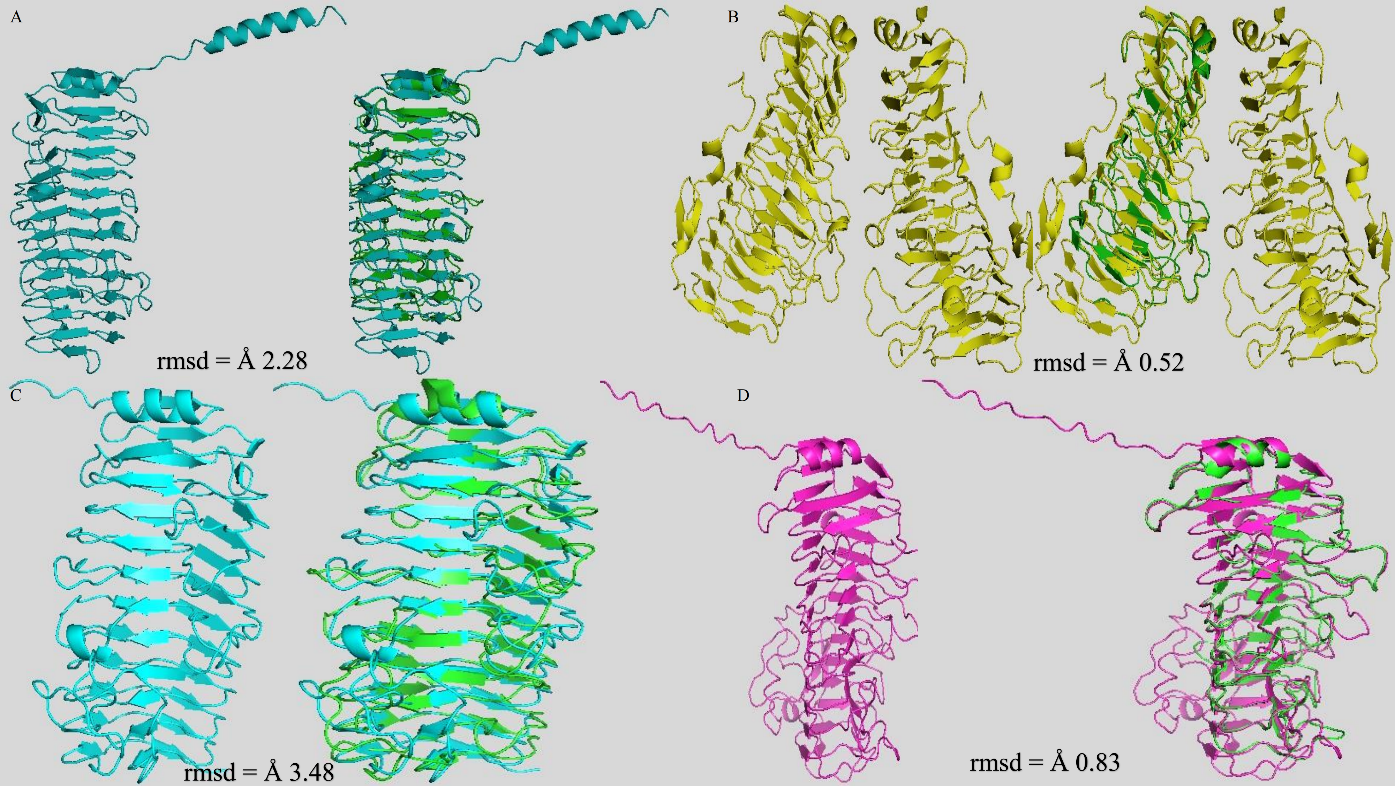


**Figure S11**. The structural comparisons of PL6 alginate lyase from our dataset with experimental structures of bacterial homologs. (A) The predicted PL6 alginate lyase (green) is superimposed with poly (beta-D-mannuronate) lyase from *Muscilaginibacter* sp. K268B (blue, PDB ID: AF-A0A1H0XFX7-F1-v4, rmsd = Å 2.28). (B) The predicted structure (green) is compared with PL6 alginate lyase from *Pseudopedobacter saltans* DSM 12145 PDB ID: 7O7A (yellow, PDB ID: 7O7A rmsd = Å 0.52). (C) The predicted structure (cyan) is aligned with a chondroitinase B-like protein from *Filimonas* sp. YR581 (blue, PDB ID: AF-A0A2U1A2B6-F1-v4 rmsd = Å 3.48). (D) The predicted structure (green) is superimposed with an alginate lyase from *Ignavibacteriales* bacterium (purple, PDB ID: AF-A0A419H0L1-F1-v4 rmsd = Å 0.83). Structural alignments were performed using US-align to superimpose the models, and diagrams were visualized in PyMOL, with alpha helices, beta sheets, and loops depicted in distinct colors for predicted and experimental structures.


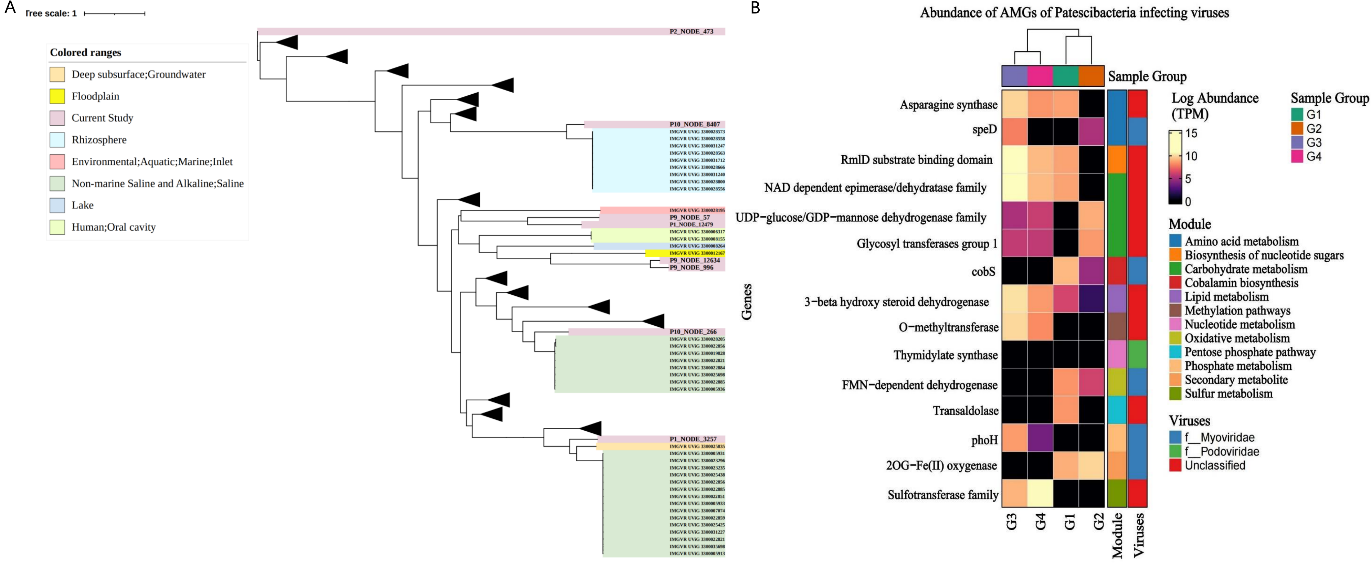


**Figure S12**. (A) Maximum-likelihood phylogenetic tree of Patescibacteria-infecting viruses based on the TerL protein sequence, constructed using IQ-TREE. The IMG_VR taxonomy was at the class level. Red branches indicate our contigs. (B) The heat map on the left displays the relative abundance (TPM) of detected AMGs within each group. The host phyla of the viruses carrying the detected AMGs are displayed on the right (green tiles). The color scale is shown as a log-transformed (TPM).
